# Integrating the markers Pan I and haemoglobin with the genetic linkage map of Atlantic cod (*Gadus morhua*)

**DOI:** 10.1186/1756-0500-3-261

**Published:** 2010-10-15

**Authors:** Tudor Borza, Brent Higgins, Gary Simpson, Sharen Bowman

**Affiliations:** 1 Genome Atlantic, NRC Institute for Marine Biosciences, Halifax, NS, Canada

## Abstract

**Background:**

Haemoglobin (Hb) and pantophysin (Pan I) markers have been used intensively in population studies of Atlantic cod (*Gadus morhua*) and in the analysis of traits such as temperature tolerance, growth characteristics and sexual maturation. We used an Illumina GoldenGate panel and the KASPar SNP genotyping system to analyse SNPs in three Atlantic cod families, one of which was polymorphic at the Hb β1 locus, and to generate a genetic linkage map integrating Pan I and multiple Hb loci.

**Findings:**

Data generated allowed the mapping of nine Hb loci, the Pan I locus, and other 122 SNPs onto an existing linkage genetic map for Atlantic cod. Four Hb genes (i.e. α1, α4, β1 and β5) have been mapped on linkage group (LG) 2 while the other five (i.e. α2, α3, β2, β3 and β4) were placed on LG18. Pan I was mapped on LG 1 using a newly developed KASPar assay for a SNP variable only in Pan I^A ^allelic variants. The new linkage genetic map presented here comprises 1046 SNPs distributed between 23 linkage groups, with a length of 1145.6 cM. A map produced by forcing additional loci, resulting in a reduced goodness-of-fit for mapped markers, allowed the mapping of a total of 1300 SNPs. Finally, we compared our genetic linkage map data with the genetic linkage map data produced by a different group and identified 29 shared SNPs distributed on 10 different linkage groups.

**Conclusions:**

The genetic linkage map presented here incorporates the marker Pan I, together with multiple Hb loci, and integrates genetic linkage data produced by two different research groups. This represents a useful resource to further explore if Pan I and Hbs or other genes underlie quantitative trait loci (QTL) for temperature sensitivity/tolerance or other phenotypes.

## Findings

The Atlantic cod (*Gadus morhua*) represents one of the most valuable commercial resources for international fisheries [1]. Haemoglobin (Hb), pantophysin (Pan I), and microsatellite markers have been widely used to characterize the genetic diversity of Atlantic cod populations and their dispersal characteristics [2,3]. In addition, Hb analyses have revealed correlations between allele types and traits such as growth [2,4] , water temperature preference [5], age and seasonality of sexual maturation [6] or annual mortality [7]. Similarly, growth rate [8], water temperature, salinity and depth appear to have an effect on Pan I allele frequencies [2,9,10]. Recently, a large number of single nucleotide polymorphisms (SNPs) have been identified [11-13] and two independent genetic linkage maps have been generated [12,14]. However, although SNPs have been characterized in both Hb [15,16] and Pan I [9,17,18], the position of these markers on a linkage map has yet to be determined. To overcome this deficit we used a combination of methods, i.e. a Illumina GoldenGate panel (Illumina Inc.) comprising 1536 SNPs and the KASPar SNP genotyping system (KBioscience, UK) to place these genes onto a genetic linkage map.

### Mapping the haemoglobin genes

**Figure 1:**
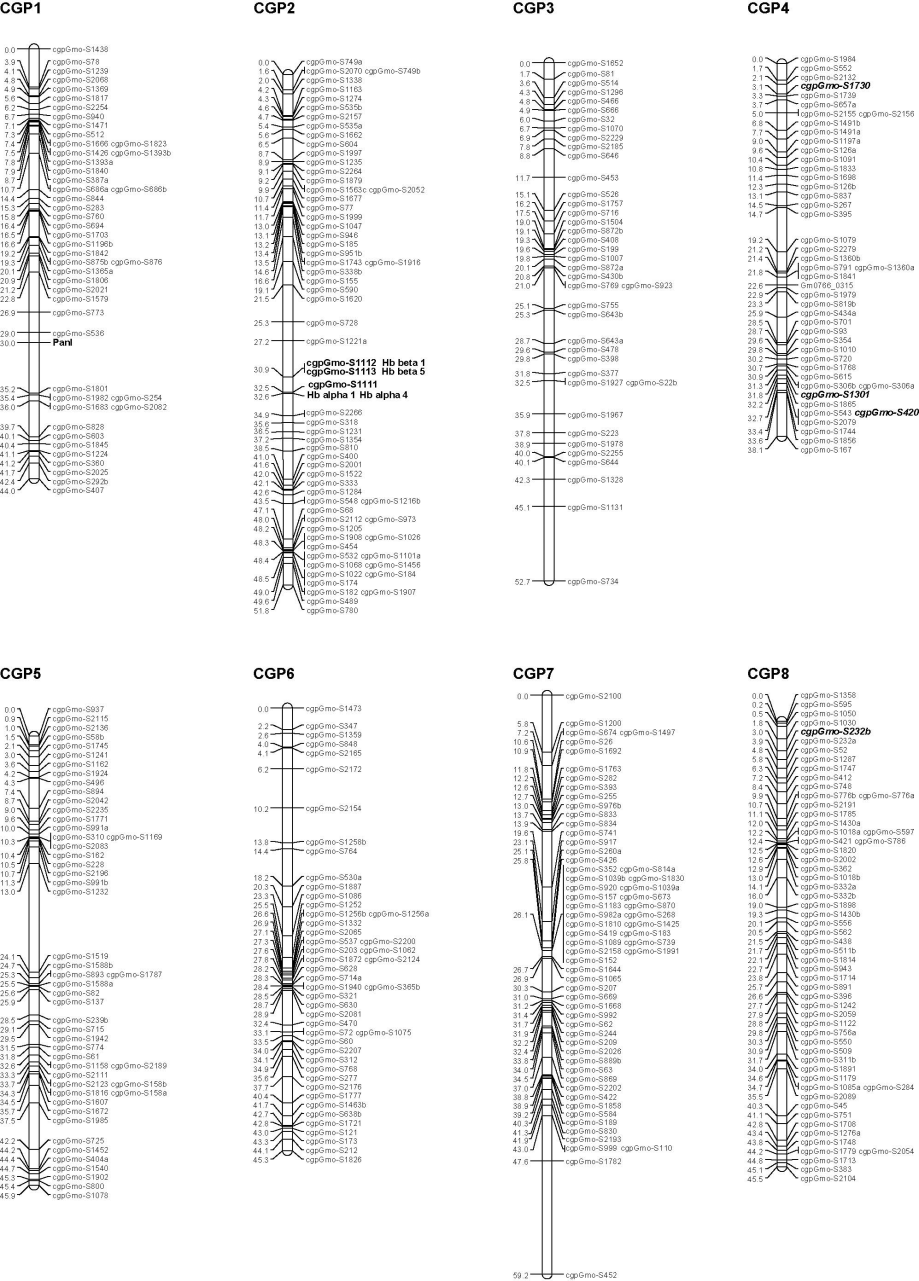
**Genetic linkage map for Atlantic cod (linkage groups 1-8)**. The first eight of the 23 major linkage groups are shown. The 23 major linkage groups have been numbered CGP 1 to 23 as in Hubert *et al. *2010, to distinguish them from the linkage groups generated by Moen *et al. *2009. Distances in centimorgans (Kosambi cM) are indicated on the left of each linkage group, with SNP identifiers on the right. Pan I and Hb loci are highlighted in bold, while SNPs common to Moen *et al. *2009 are both bold and italicized.

**Figure 2:**
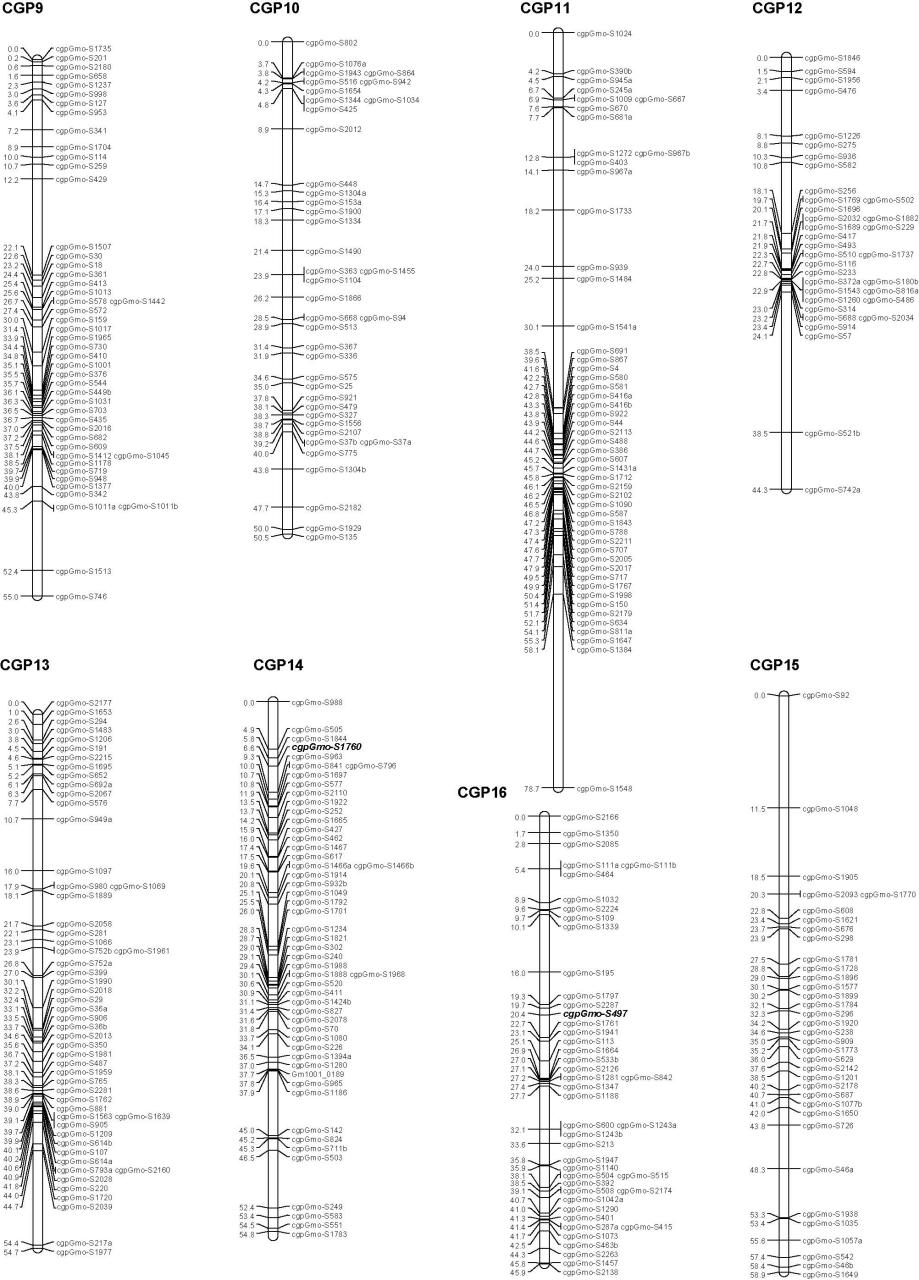
**Genetic linkage map for Atlantic cod (linkage groups 9-16)**. Eight of the 23 major linkage groups are shown. The 23 major linkage groups have been numbered CGP 1 to 23 as in Hubert *et al. *2010, to distinguish them from the linkage groups generated by Moen *et al. *2009. Distances in centimorgans (Kosambi cM) are indicated on the left of each linkage group, with SNP identifiers on the right. SNPs common to Moen *et al. *2009 are highlighted in bold and italicized.

**Figure 3:**
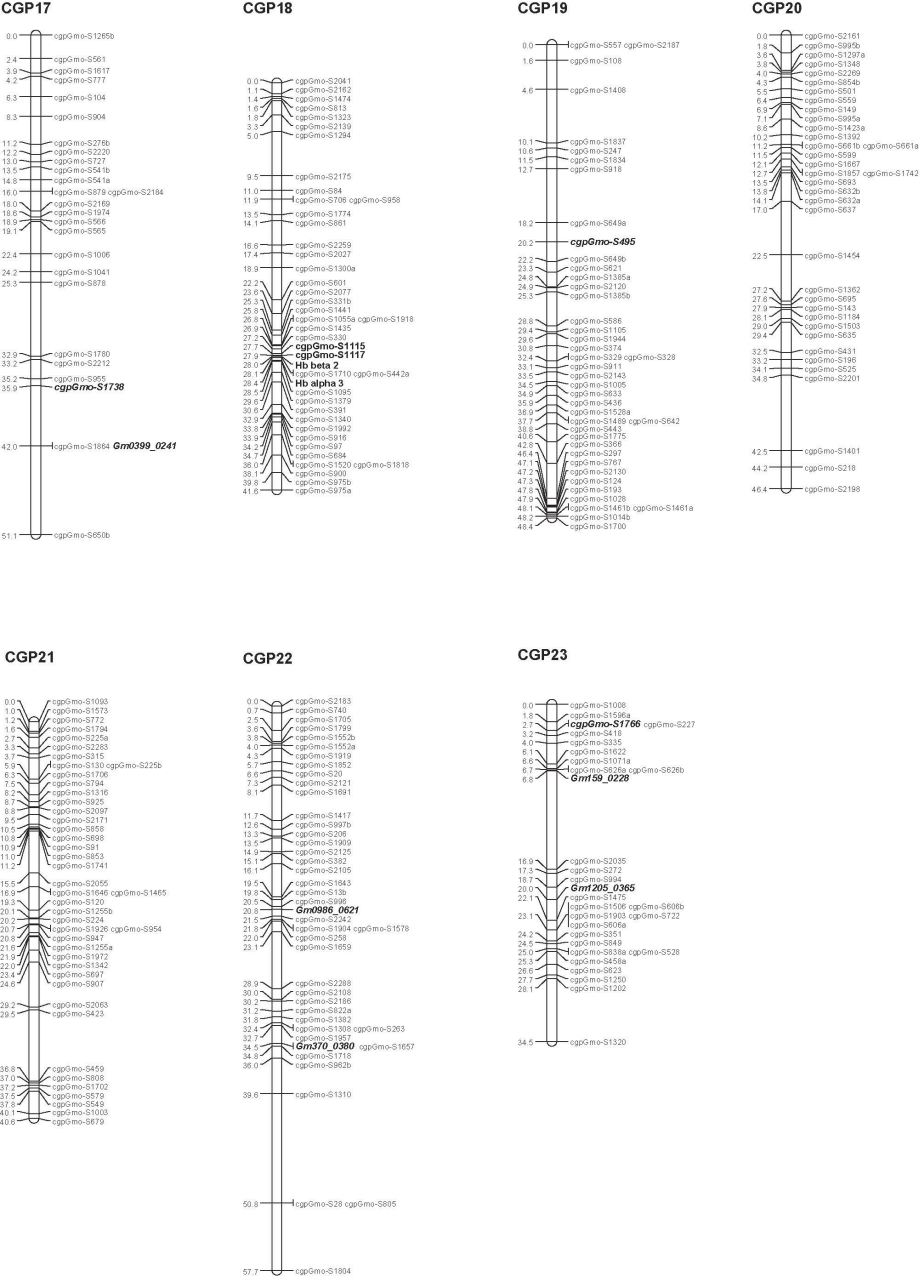
**Genetic linkage map for Atlantic cod (linkage groups 17-23)**. Seven of the 23 major linkage groups are shown. The 23 major linkage groups have been numbered CGP 1 to 23 as in Hubert *et al. *2010, to distinguish them from the linkage groups generated by Moen *et al. *2009. Distances in centimorgans (Kosambi cM) are indicated on the left of each linkage group, with SNP identifiers on the right. Hb loci are highlighted in bold while SNPs common to Moen *et al. *2009 are both bold and italicized.

We used a curated Illumina GoldenGate panel containing 1536 SNPs [12], including three SNPs identified in the Hb β1 gene, two SNPs specific to the Hb β3 gene and one characteristic for the Hb β4 gene (details in Additional file 1). These SNPs were derived from polymorphisms detected by sequencing nine Atlantic cod Hb genes in the parents and 15 progeny of a family, B30, that was determined to be heterozygous (1/2) at the HbI locus [16]. Western Atlantic cod populations are characterized by a low frequency of allele 1 at the HbI locus [19,20]. Family B30 was one of the few families heterozygous at this locus produced by the breeding program of Atlantic Cod Genomics and Broodstock Development Project (CGP) [11]; parents and 93 progeny from this family were used to map these loci. Illumina GoldenGate probe design failed for several of the Hb SNPs, therefore additional Hb polymorphisms were identified and used to develop assays for the KASPar SNP genotyping system (Additional file 2). A genetic linkage map was constructed using JoinMap^®^4 [21]. The genotypes for progeny were converted to CP codes based on parental genotypes and mapping was performed using a LOD cut-off value of 5.0 and Kosambi's mapping function [21]. The 23 linkage groups obtained for family B30 were compared to the linkage groups previously reported by Hubert *et al. *2010 [12]; a 1:1 correspondence between linkage groups (LGs) was confirmed and the corresponding LGs were merged (Figures 1, 2, 3; more details about mapping can be found in Hubert *et al. *2010 and Additional file 1). The 23 linkage groups observed for family B30 (and those described in Hubert *et al. *2010) matches the number of haploid chromosomes reported for the Atlantic cod by Fan and Fox [22]. The combined results from GoldenGate and KASPar assays allowed the mapping of all Hb genes analysed: Hb genes α1, α4, β1 and β5 were mapped on LG 2 while Hb genes α3 and β2, β3 and β4 were mapped on LG 18 (Figures 1 and 3). Sequencing data from the Hb α2 gene, which was not polymorphic in the B30 family, indicate that this gene is situated immediately adjacent to the Hb β3 gene [23,24]; therefore the cluster of Hb genes located on LG 18 contains at least five different Hb genes. The mapping of Atlantic cod Hb genes is in agreement with other studies on fish and other vertebrates which indicate that Hb loci are placed on two linkage groups/two different chromosomes [25,26]. Allele frequencies at the Hb β1 locus from several North Atlantic cod populations (Additional file 2) were similar to data collected by Hb allozyme electrophoresis [19,20] suggesting that Hb β1, or other closely linked genes, might underlie QTLs.

### Mapping Pan I

The two main allele variants Pan I^A ^and Pan I^B ^described at the Pan locus can be determined by assessing the polymorphism present at a DraI site located in intron 4 [9,17,27]. However, these alleles have very different frequencies within different Atlantic cod stocks, and all candidate families from the CGP [11] have been determined to be Pan I^A ^homozygotes (Additional file 2). To overcome this problem a new assay was developed using the KASPar SNP genotyping system and the non-synonymous substitution (Glu/Lys) present in exon 4 (G767A), which is variable only in Pan I^A ^allelic variants [17]. Family B87, one of the two families used in the generation of the initial map [12] was determined to be polymorphic for Pan I^A ^G767A and the Pan I locus was mapped on LG1 (Figure 1; Additional files 1 and 2) using that family.

### The addition of family B30 allowed new SNPs to be mapped onto the CGP map

The new map, resulted from the addition of family B30, and of Pan I and Hb loci, contains 1046 mapped SNPs, an increase of 122 SNPs when compared to the genetic linkage map reported earlier by Hubert *et al. *2010 [12]. This map was generated by the first round of calculations of the regression mapping algorithm and the positions of all loci were statistically strongly supported [21]. Another map, produced by the third round of mapping calculations [21] integrates 1300 SNPs, which represents a contribution of 376 new SNPs that could be mapped (Additional file 3). This map was generated to obtain a general idea of where poorer fitting loci reside on the genetic linkage map [21].

### Linkage group correspondence between maps produced by independent research groups

Finally, we aimed to merge the CGP genetic linkage map data (this paper and Hubert *et al. *[12]) with the genetic linkage map data produced by a different group [14]. To identify sequences that were mapped by both, CGP (this paper and Hubert *et al. *[12]) and Moen *et al. *[14] we performed a Blastn search of an in-house made database containing the ESTs listed by Moen *et al. *[14] using 124 nucleotides long CGP SNP-containing sequences. EST sequence data used by Moen *et al. *was retrieved from GenBank using the GenBank accession numbers listed by this group [14]. Using this approach we identified 29 shared SNPs distributed on 10 different linkage groups (Additional file 4). The minimal overlap between the linkage map produced in the present study and that of Moen *et al. *[14] might result from ascertainment bias caused by different frequencies of SNPs in NE and NW Atlantic populations of cod and/or because CGP sequenced the 3' ends of ESTs to identify SNPs [11,12] whereas Moen *et al. *[13,14] sequenced the 5' ends of their ESTs to detect polymorphisms.

## Conclusions

The genetic linkage map presented here, that includes the marker PanI and multiple Hb loci, represents a useful resource for studying genotype-phenotype relationships, for QTL studies, as well as for population studies. Our data indicate that Hb genes are located on two different linkage groups while Pan I locus was mapped on a third linkage group. Further studies are needed to elucidate which of these genes/linkage groups will correlate with phenotypic traits. The Hb β1 gene, which has been linked to variation in haemoglobin oxygen binding capacity and water temperature preference [15,28] (although the role of this gene in temperature adaptation is still subject to debate [16]) was mapped on LG 2. New avenues for physiological and biochemical studies related to temperature and hypoxia tolerance in Atlantic cod and other fishes may result if, through further studies, it can be demonstrated that this gene is associated with relevant QTL.

## Competing interests

The authors declare that they have no competing interests.

## Authors' contributions

TB initiated the analysis of family B30 using the GoldenGate panel and the KASPar SNP genotyping system, selected SNPs for the mapping of PanI and Hb loci, generated the genetic linkage map and wrote the paper. BH wrote custom Perl scripts which were used for analysis of the data generated from the GoldenGate panel, and performed SNP analysis using the KASPar SNP genotyping system. GS performed SNP analysis using the KASPar SNP genotyping system. SB was responsible for the conceptualization, design and implementation of the SNP identification and analysis program and edited the final versions of the manuscript. All authors read and approved the final manuscript.

## Supplementary Material

Additional file 1 **Properties of the SNPs for which GoldenGate or KASPar functional assay was developed**. This file includes SNP locus name, SNP accession number, position on the linkage group, sequence information regarding the SNP and the flanking regions, SNP annotation and the results of the tests for departure from Mendelian segregation. Click here for file

Additional file 2 **Primer sequence used for the KASPar SNP genotyping system and details regarding the SNPs used to map Hb β1 and Pan I**. This file contains the list of primers designed for the KASPar SNP genotyping system, and the genotyping results obtained by screening several Atlantic cod populations with the Hb β1 and PanI SNPs used in the mapping process. Click here for file

Additional file 3 **Genetic linkage map with forced additional loci**. This file contains the genetic linkage map with forced additional loci. The 23 major linkage groups have been numbered CGP 1 to 23 as in Hubert *et al. *2010, to distinguish them from the linkage groups generated by Moen *et al. *2009. Distances in centimorgans (Kosambi cM) are indicated on the left of each linkage group, with SNP identifiers on the right. PanI and Hb loci are in bold, italicized and highlighted in red while SNPs common to Moen *et al. *2009 are in bold and highlighted in red. Click here for file

Additional file 4 **Linkage group correspondence between the genetic linkage groups presented in this study and Moen *et al. *2009**. This file contains the data related to the linkage group correspondence between the genetic linkage groups presented in this study and Moen *et al. *2009. Click here for file
