# Supplementary material for: Integrating the markers Pan I and haemoglobin with the genetic linkage map of Atlantic cod (Gadus morhua)
Source: BMC Res Notes. 2010 Oct 15;3:261. doi: 10.1186/1756-0500-3-261 (PMC3020663; doi:10.1186/1756-0500-3-261)
Supplement: Additional file 2 — Primer sequence used for the KASPar SNP genotyping system and details regarding the SNPs used to map Hb β1 and Pan I. This file contains the list of primers designed for the KASPar SNP genotyping system, and the genotyping results obtained by screening several Atlantic cod populations with the Hb β1 and PanI SNPs used in the mapping process. [file 1756-0500-3-261-S2.DOC]

**Additional file 2.**

**1. KASPar SNP genotyping system primers**

1.1. Primers used in genotyping the hemoglobin genes. The position of these SNPs can be found in Appendix S1 and in [1].

Hb α1

A1SNP2ALC **GAAGGTGACCAAGTTCATGCT**AATTGTTCAAGTTATTCCCCCTAACTG

A1SNP2ALG **GAAGGTCGGAGTCAACGGATT**AATTGTTCAAGTTATTCCCCCTAACTC

A1SNP2C1 GCCGATGCTCTTTCAAGGTATGCTT

Hb α3

A3SNP1ALC **GAAGGTGACCAAGTTCATGCT**CATATGCCTACAGGTCTACATGC

A3SNP1ALT **GAAGGTCGGAGTCAACGGATT**GCATATGCCTACAGGTCTACATGT

A3SNP1C1 CTCATATACCTAGACAAACCTTWGTGTATA

Hb α4

A4SNP3ALA **GAAGGTGACCAAGTTCATGCT**CCTTTTATTGATCGTTATTTTACACCTGAAC

A4SNP3ALT **GAAGGTCGGAGTCAACGGATT**CCTTTTATTGATCGTTATTTTACACCTGAA**G**

A4SNP3C1 TGCGCATTGACCCTGCAAACTTCAA

Hb β1

B1ALA **GAAGGTGACCAAGTTCATGCT**GGGCCACGACGCCGTGCT

B1ALC **GAAGGTCGGAGTCAACGGATT**GGCCACGACGCCGTGCG

B1C1 GCCGCTATTRTGGGAAACCCCAA

Hb β2

B2ALG **GAAGGTGACCAAGTTCATGCT**GACAGACAGGAACTTCTGCCAC

B2ALA **GAAGGTCGGAGTCAACGGATT**CGACAGACAGGAACTTCTGCCAT

B2C1 CAAATTCACCGTGGAGACCCAGGT

Hbβ5

B5ALG **GAAGGTGACCAAGTTCATGCT**AAATTAGACACGTTTTAATGGGAATGTTTG

B5ALT **GAAGGTCGGAGTCAACGGATT**ATAAATTAGACACGTTTTAATGGGAATGTTTT

B5C1 GGCGATTGCAAGGCAGTCAGCAA

1.2. Primers used in genotyping the PanI gene

DraI_PanI^A^ **GAAGGTCGGAGTCAACGGATT**AGAAAAATGTCTCAGTTCCCCATTTTG

DraI_PanI^B^ **GAAGGTGACCAAGTTCATGCT**CAGAAAAATGTCTCAGTTCCCCATTTTA

DraI_S CAACGTGGGTTCAAGCTTAAGCAGAT

PAB_ALA **GAAGGTGACCAAGTTCATGCT**GACTYAGTGGTGCCATTCTTACAGT

PAB_ALC **GAAGGTCGGAGTCAACGGATT**CTYAGTGGTGCCATTCTTACAGG

PAB_C1 GTTTCTCCTACTTTAGGTTGATGC

PKA_ALA **GAAGGTGACCAAGTTCATGCT**GTAGTTGCCAATAAGGAAAGACTT

PKA_ALG **GAAGGTCGGAGTCAACGGATT**GTAGTTGCCAATAAGGAAAGACTC

PKA_C1 GTTTCTCCTACTTTAGGTTGATGC

PAB= C747/753A site; PKA= G767A site (see below)

**2. SNPs used to map the Hb β1 gene on the genetic linkage map.** Several SNPs were assessed for this gene in order to determine whether they are present in individuals with different geographic localization. More details related to SNP position and selection can be found in [1]. Details regarding the Atlantic cod population used for screening the Hb β1 gene SNPs can be found in [2] and [3]. The SNP T21C (cgpGmo-S1112) that was used in the B30 family mapping was monomorphic (T) in all populations tested.

|  | Number of individuals | A740T  (cgpGmo-S1113)  Illumina GoldenGate technology | | | SNP G454A; Lys/Ala  KASPar SNP genotyping system | | | SNP A1092T; Leu/Met (cgpGmo-S1111)  Illumina GoldenGate technology | | |
| --- | --- | --- | --- | --- | --- | --- | --- | --- | --- | --- |
|  |  | **AA** | **AB** | **BB** | **AA** | **AB** | **BB** | **AA** | **AB** | **BB** |
| Galway Bay, Ireland | **15** | 7 | 5 | 3 | 7 | 5 | 3 | 15 | 0 | 0 |
| Barents Sea, Norway | **25/26** | 0 | 5 | 20 | 0 | 6 | 20 | 24 | 1 | 0 |
| Akureyri, Iceland | **26** | 0 | 1 | 25 | 0 | 2 | 24 | 20 | 4 | 2 |
| Bay Bulls, NL, Canada | **23** | 0 | 9 | 14 | 0 | 6 | 17 | 1 | 9 | 13 |
| Smith Sound, NL, Canada | **23** | 0 | 0 | 23 | 0 | 5 | 18 | 0 | 12 | 11 |
| Georges Bank, NB, Canada | **23/24** | 0 | 5 | 19 | 0 | 6 | 17 | 4 | 13 | 7 |
| Cape Sable, NS, Canada | **22/23** | 0 | 6 | 17 | 0 | 7 | 15 | 4 | 12 | 7 |

**3. SNPs used to map the PanI gene on the genetic linkage map.** The two main allele variants PanI^A^ and PanI^B^ described at the Pan locus can be determined by assessing the polymorphism present at a DraI site (a G/A substitution in intron 4. Allele A: DraI site absent - TTTTGAAA; Allele B DraI site present - TTTTAAAA)[4-7]. However, the frequency of PanI^B^ is low or zero in Canadian Atlantic cod populations (see below) and all families used for mapping [8, 9] were PanI^A^ /PanI^A^ (data not shown); therefore a different polymorphic site (G767A site) [5] was used to map PanI gene. Our screening also indicated that another polymorphic site, C747/753A [5], which generate a non-synonymous substitution, can be used as an alternative of the DraI site.

|  | Number of individuals | G1132/1138A; DraI site  GenBank acc.#  (AF288943)/(AF288970) | | | C747/753A site  A**C**C – Thr(T)/G**A**C – Asp (D); linked to the DraI site  GenBank acc.#  (AF288943)/(AF288970) | | | | G767A site  **G**AG – Glu(E)/**A**AG – Lys (K)  GenBank acc.#  (AF288943/AF288952) | | |
| --- | --- | --- | --- | --- | --- | --- | --- | --- | --- | --- | --- |
|  |  | **AA** | **AB** | **BB** | | **AA** | **AB** | **BB** | **AA** | **AB** | **BB** |
| Galway Bay, Ireland | **15** | 15 | 0 | 0 | | 15 | 0 | 0 | 8 | 6 | 1 |
| Barents Sea, Norway | **26** | 0 | 5 | 21 | | 0 | 5 | 21 | 24 | 2 | 0 |
| Akureyri, Iceland | **26** | 26 | 0 | 0 | | 26 | 0 | 0 | 18 | 6 | 2 |
| Bay Bulls, NL, Canada | **20** | 9 | 8 | 3 | | 9 | 8 | 3 | 7 | 8 | 5 |
| Smith Sound, NL, Canada | **21** | 18 | 3 | 0 | | 18 | 3 | 0 | 3 | 9 | 9 |
| Georges Bank, NB, Canada | **23** | 23 | 0 | 0 | | 23 | 0 | 0 | 9 | 8 | 6 |
| Cape Sable, NS, Canada | **23** | 23 | 0 | 0 | | 23 | 0 | 0 | 4 | 13 | 6 |

**References**

1. Borza T, Stone C, Gamperl AK, Bowman S: **Atlantic cod (*Gadus morhua*) hemoglobin genes: multiplicity and polymorphism**. *BMC Genet* 2009, **10**:51.

2. Bowman S, Hubert S, Higgins B, Stone C, Kimball J, Borza T, Tarrant Bussey J, Simpson G, Kozera C, Curtis BA *et al*: **An integrated approach to gene discovery and marker development in Atlantic cod (*Gadus morhua*)**. *Marine Biotechnology* 2010, **in press**.

3. Hubert S, Higgins B, Borza T, Bowman S: **Development of a SNP resource and a genetic linkage map for Atlantic cod (*Gadus morhua*)** *BMC Genomics* 2010, **11**.

4. Stenvik J, Wesmajervi MS, Damsgard B, Delghandi M: **Genotyping of pantophysin I (Pan I) of Atlantic cod (Gadus morhua L.) by allele-specific PCR**. *Mol Ecol Notes* 2006, **6**(1):272-275.

5. Pogson GH: **Nucleotide polymorphism and natural selection at the pantophysin (Pan I) locus in the Atlantic cod, *Gadus morhua* (L.)**. *Genetics* 2001, **157**(1):317-330.

6. Pogson GH, Fevolden S-E: **Natural selection and the genetic differentiation of coastal and Arctic populations of the Atlantic cod in northern Norway: a test involving nucleotide sequence variation at the pantophysin (Pan) locus**. *Molecular Ecology* 2003, **12**(1):63-74.

7. Fevolden SE, Pogson GH: **Genetic divergence at the synaptophysin (Syp I) locus among Norwegian coastal and north-east Arctic populations of Atlantic cod** *Journal of Fish Biology* 1997, **51**(5):895-908.

8. Bowman S, Hubert S, Higgins B, Stone C, Kimball J, Borza T, Bussey JT, Simpson G, Kozera C, Curtis BA *et al*: **An integrated approach to gene discovery and marker development in Atlantic cod (*Gadus morhua*)**. *Mar Biotechnol (NY)* 2010.

9. Hubert S, Higgins B, Borza T, Bowman S: **Development of a SNP resource and a genetic linkage map for Atlantic cod (*Gadus morhua*)**. *BMC Genomics* 2010, **11**:191.
