# Supplementary material for: Integrating the markers Pan I and haemoglobin with the genetic linkage map of Atlantic cod (Gadus morhua)
Source: BMC Res Notes. 2010 Oct 15;3:261. doi: 10.1186/1756-0500-3-261 (PMC3020663; doi:10.1186/1756-0500-3-261)
Supplement: Additional file 3 — Genetic linkage map with forced additional loci. This file contains the genetic linkage map with forced additional loci. The 23 major linkage groups have been numbered CGP 1 to 23 as in Hubert et al. 2010, to distinguish them from the linkage groups generated by Moen et al. 2009. Distances in centimorgans (Kosambi cM) are indicated on the left of each linkage group, with SNP identifiers on the right. PanI and Hb loci are in bold, italicized and highlighted in red while SNPs common to Moen et al. 2009 are in bold and highlighted in red. [file 1756-0500-3-261-S3.PDF]

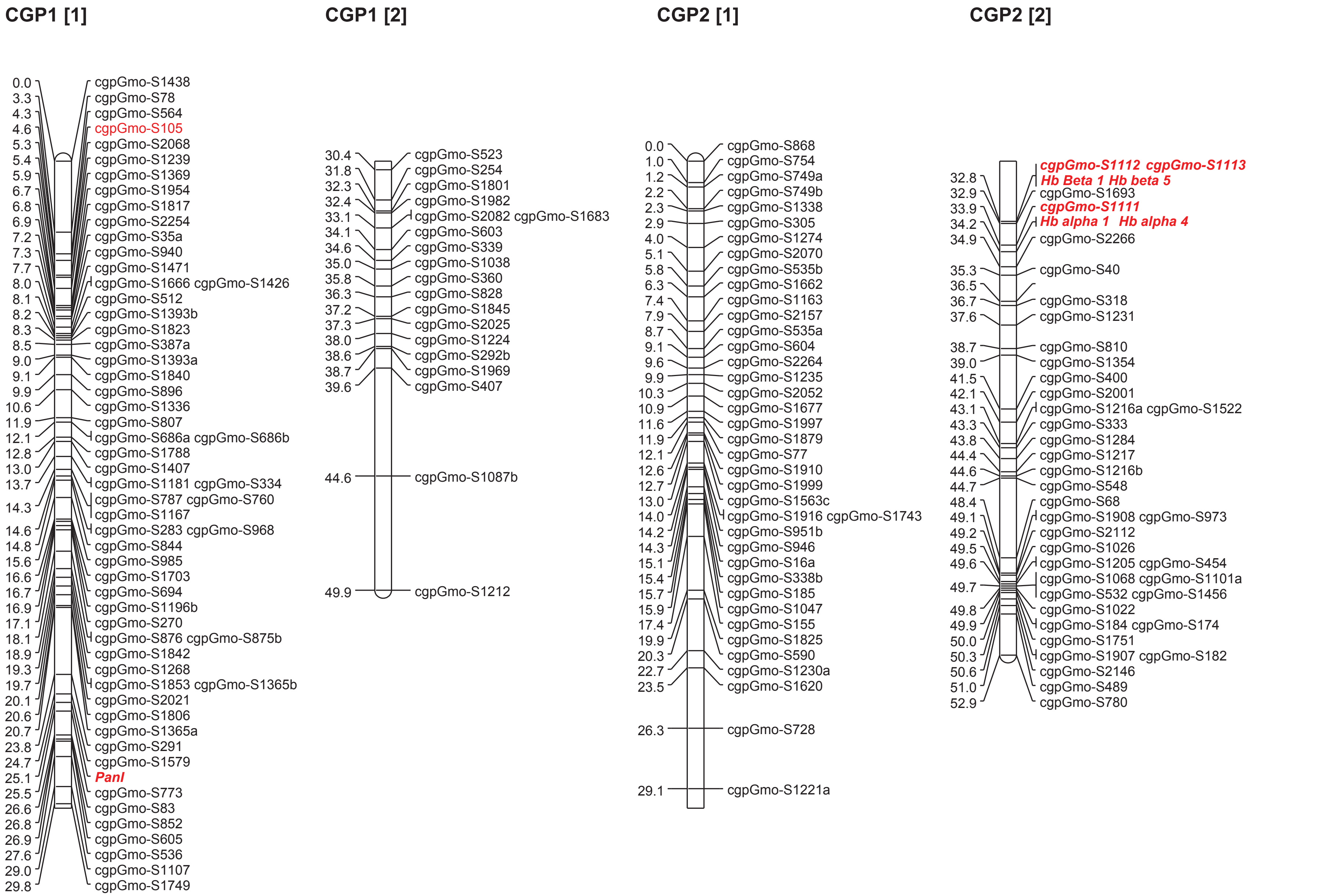
CGP1 [1]
0.0
3.3
4.3
4.6
5.3
5.4
5.9
6.7
6.8
6.9
7.2
7.3
7.7
8.0
8.1
8.2
8.3
8.5
9.0
9.1
9.9
10.6
11.9
12.1
12.8
13.0
13.7
14.3
14.6
14.8
15.6
16.6
16.7
16.9
17.1
18.1
18.9
19.3
19.7
20.1
20.6
20.7
23.8
24.7
25.1
25.5
26.6
26.8
26.9
27.6
29.0
29.8
cgpGmo-S1438
cgpGmo-S78
cgpGmo-S564
cgpGmo-S105
cgpGmo-S2068
cgpGmo-S1239
cgpGmo-S1369
cgpGmo-S1954
cgpGmo-S1817
cgpGmo-S2254
cgpGmo-S35a
cgpGmo-S940
cgpGmo-S1471
cgpGmo-S1666 cgpGmo-S1426
cgpGmo-S512
cgpGmo-S1393b
cgpGmo-S1823
cgpGmo-S387a
cgpGmo-S1393a
cgpGmo-S1840
cgpGmo-S896
cgpGmo-S1336
cgpGmo-S807
cgpGmo-S686a cgpGmo-S686b
cgpGmo-S1788
cgpGmo-S1407
cgpGmo-S1181 cgpGmo-S334
cgpGmo-S787 cgpGmo-S760
cgpGmo-S1167
cgpGmo-S283 cgpGmo-S968
cgpGmo-S844
cgpGmo-S985
cgpGmo-S1703
cgpGmo-S694
cgpGmo-S1196b
cgpGmo-S270
cgpGmo-S876 cgpGmo-S875b
cgpGmo-S1842
cgpGmo-S1268
cgpGmo-S1853 cgpGmo-S1365b
cgpGmo-S2021
cgpGmo-S1806
cgpGmo-S1365a
cgpGmo-S291
cgpGmo-S1579
PanI
cgpGmo-S773
cgpGmo-S83
cgpGmo-S852
cgpGmo-S605
cgpGmo-S536
cgpGmo-S1107
cgpGmo-S1749
CGP1 [2]
30.4
31.8
32.3
32.4
33.1
34.1
34.6
35.0
35.8
36.3
37.2
37.3
38.0
38.6
38.7
39.6
44.6
49.9
cgpGmo-S523
cgpGmo-S254
cgpGmo-S1801
cgpGmo-S1982
cgpGmo-S2082 cgpGmo-S1683
cgpGmo-S603
cgpGmo-S339
cgpGmo-S1038
cgpGmo-S360
cgpGmo-S828
cgpGmo-S1845
cgpGmo-S2025
cgpGmo-S1224
cgpGmo-S292b
cgpGmo-S1969
cgpGmo-S407
cgpGmo-S1087b
cgpGmo-S1212
CGP2 [1]
0.0
1.0
1.2
2.2
2.3
2.9
4.0
5.1
5.8
6.3
7.4
7.9
8.7
9.1
9.6
9.9
10.3
10.9
11.6
11.9
12.1
12.6
12.7
13.0
14.0
14.2
14.3
15.1
15.4
15.7
15.9
17.4
19.9
20.3
22.7
23.5
26.3
29.1
cgpGmo-S868
cgpGmo-S754
cgpGmo-S749a
cgpGmo-S749b
cgpGmo-S1338
cgpGmo-S305
cgpGmo-S1274
cgpGmo-S2070
cgpGmo-S535b
cgpGmo-S1662
cgpGmo-S1163
cgpGmo-S2157
cgpGmo-S535a
cgpGmo-S604
cgpGmo-S2264
cgpGmo-S1235
cgpGmo-S2052
cgpGmo-S1677
cgpGmo-S1997
cgpGmo-S1879
cgpGmo-S77
cgpGmo-S1910
cgpGmo-S1999
cgpGmo-S1563c
cgpGmo-S1916 cgpGmo-S1743
cgpGmo-S951b
cgpGmo-S946
cgpGmo-S16a
cgpGmo-S338b
cgpGmo-S185
cgpGmo-S1047
cgpGmo-S155
cgpGmo-S1825
cgpGmo-S590
cgpGmo-S1230a
cgpGmo-S1620
cgpGmo-S728
cgpGmo-S1221a
CGP2 [2]
32.8
32.9
33.9
34.2
34.9
35.3
36.5
36.7
37.6
38.7
39.0
41.5
42.1
43.1
43.3
43.8
44.4
44.6
44.7
48.4
49.1
49.2
49.5
49.6
49.7
49.8
49.9
50.0
50.3
50.6
51.0
52.9
cgpGmo-S1112 cgpGmo-S1113
Hb Beta 1 Hb beta 5
cgpGmo-S1693
cgpGmo-S1111
Hb alpha 1 Hb alpha 4
cgpGmo-S2266
cgpGmo-S40
cgpGmo-S318
cgpGmo-S1231
cgpGmo-S810
cgpGmo-S1354
cgpGmo-S400
cgpGmo-S2001
cgpGmo-S1216a cgpGmo-S1522
cgpGmo-S333
cgpGmo-S1284
cgpGmo-S1217
cgpGmo-S1216b
cgpGmo-S548
cgpGmo-S68
cgpGmo-S1908 cgpGmo-S973
cgpGmo-S2112
cgpGmo-S1026
cgpGmo-S1205 cgpGmo-S454
cgpGmo-S1068 cgpGmo-S1101a
cgpGmo-S532 cgpGmo-S1456
cgpGmo-S1022
cgpGmo-S184 cgpGmo-S174
cgpGmo-S1751
cgpGmo-S1907 cgpGmo-S182
cgpGmo-S2146
cgpGmo-S489
cgpGmo-S780

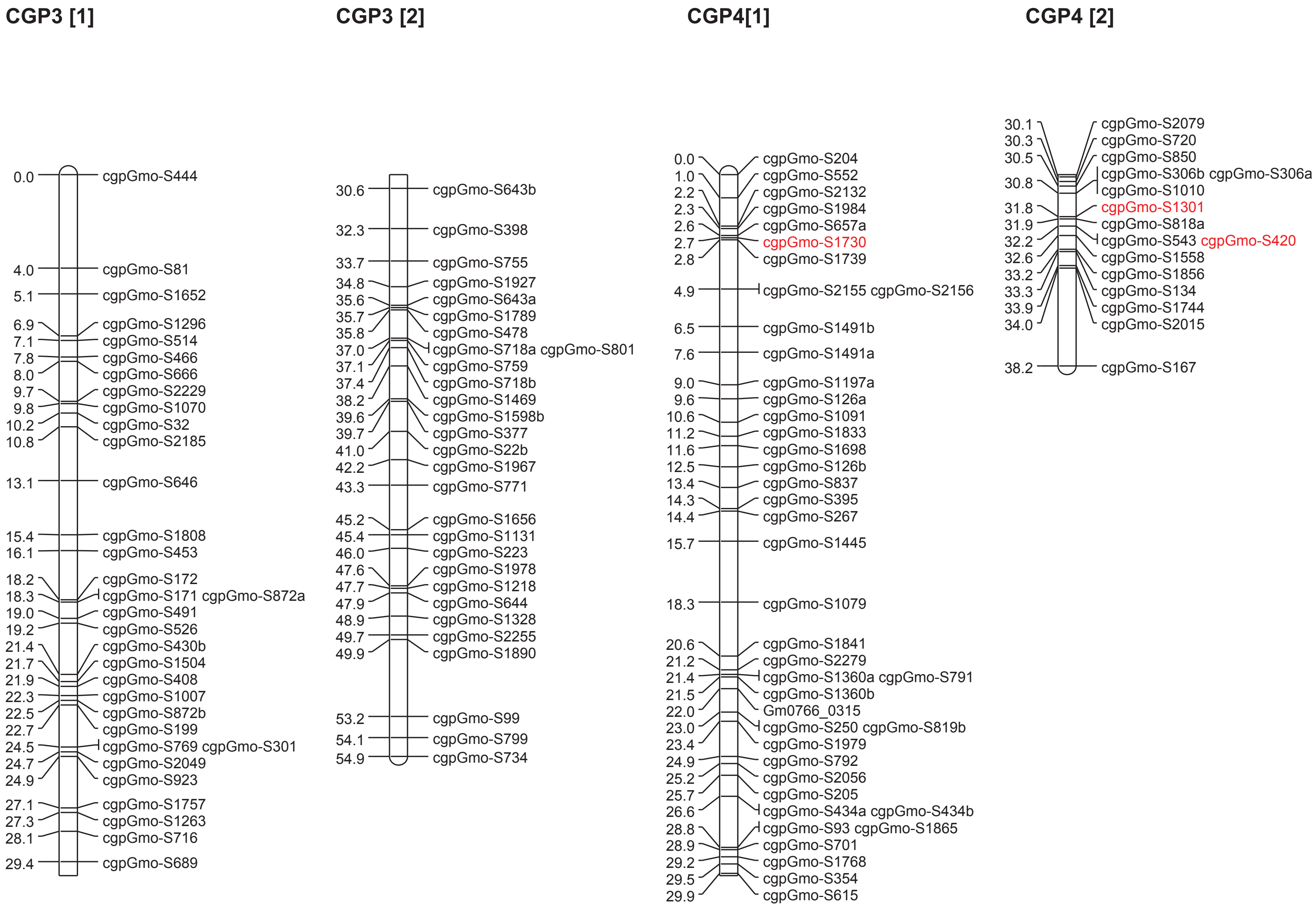
CGP3 [1]
0.0 cgpGmo-S444
4.0 cgpGmo-S81
5.1 cgpGmo-S1652
6.9 cgpGmo-S1296
7.1 cgpGmo-S514
7.8 cgpGmo-S466
8.0 cgpGmo-S666
9.7 cgpGmo-S2229
9.8 cgpGmo-S1070
10.2 cgpGmo-S32
10.8 cgpGmo-S2185
13.1 cgpGmo-S646
15.4 cgpGmo-S1808
16.1 cgpGmo-S453
18.2 cgpGmo-S172
18.3 cgpGmo-S171 cgpGmo-S872a
19.0 cgpGmo-S491
19.2 cgpGmo-S526
21.4 cgpGmo-S430b
21.7 cgpGmo-S1504
21.9 cgpGmo-S408
22.3 cgpGmo-S1007
22.5 cgpGmo-S872b
22.7 cgpGmo-S199
24.5 cgpGmo-S769 cgpGmo-S301
24.7 cgpGmo-S2049
24.9 cgpGmo-S923
27.1 cgpGmo-S1757
27.3 cgpGmo-S1263
28.1 cgpGmo-S716
29.4 cgpGmo-S689
CGP3 [2]
30.6 cgpGmo-S643b
32.3 cgpGmo-S398
33.7 cgpGmo-S755
34.8 cgpGmo-S1927
35.6 cgpGmo-S643a
35.7 cgpGmo-S1789
35.8 cgpGmo-S478
37.0 cgpGmo-S718a cgpGmo-S801
37.1 cgpGmo-S759
37.4 cgpGmo-S718b
38.2 cgpGmo-S1469
39.6 cgpGmo-S1598b
39.7 cgpGmo-S377
41.0 cgpGmo-S22b
42.2 cgpGmo-S1967
43.3 cgpGmo-S771
45.2 cgpGmo-S1656
45.4 cgpGmo-S1131
46.0 cgpGmo-S223
47.6 cgpGmo-S1978
47.7 cgpGmo-S1218
47.9 cgpGmo-S644
48.9 cgpGmo-S1328
49.7 cgpGmo-S2255
49.9 cgpGmo-S1890
53.2 cgpGmo-S99
54.1 cgpGmo-S799
54.9 cgpGmo-S734
CGP4[1]
0.0 cgpGmo-S204
1.0 cgpGmo-S552
2.2 cgpGmo-S2132
2.3 cgpGmo-S1984
2.6 cgpGmo-S657a
2.7 cgpGmo-S1730
2.8 cgpGmo-S1739
4.9 cgpGmo-S2155 cgpGmo-S2156
6.5 cgpGmo-S1491b
7.6 cgpGmo-S1491a
9.0 cgpGmo-S1197a
9.6 cgpGmo-S126a
10.6 cgpGmo-S1091
11.2 cgpGmo-S1833
11.6 cgpGmo-S1698
12.5 cgpGmo-S126b
13.4 cgpGmo-S837
14.3 cgpGmo-S395
14.4 cgpGmo-S267
15.7 cgpGmo-S1445
18.3 cgpGmo-S1079
20.6 cgpGmo-S1841
21.2 cgpGmo-S2279
21.4 cgpGmo-S1360a cgpGmo-S791
21.5 cgpGmo-S1360b
22.0 Gm0766_0315
23.0 cgpGmo-S250 cgpGmo-S819b
23.4 cgpGmo-S1979
24.9 cgpGmo-S792
25.2 cgpGmo-S2056
25.7 cgpGmo-S205
26.6 cgpGmo-S434a cgpGmo-S434b
28.8 cgpGmo-S93 cgpGmo-S1865
28.9 cgpGmo-S701
29.2 cgpGmo-S1768
29.5 cgpGmo-S354
29.9 cgpGmo-S615
CGP4 [2]
30.1 cgpGmo-S2079
30.3 cgpGmo-S720
30.5 cgpGmo-S850
30.8 cgpGmo-S306b cgpGmo-S306a
cgpGmo-S1010
31.8 cgpGmo-S1301
31.9 cgpGmo-S818a
32.2 cgpGmo-S543 cgpGmo-S420
32.6 cgpGmo-S1558
33.2 cgpGmo-S1856
33.3 cgpGmo-S134
33.9 cgpGmo-S1744
34.0 cgpGmo-S2015
38.2 cgpGmo-S167

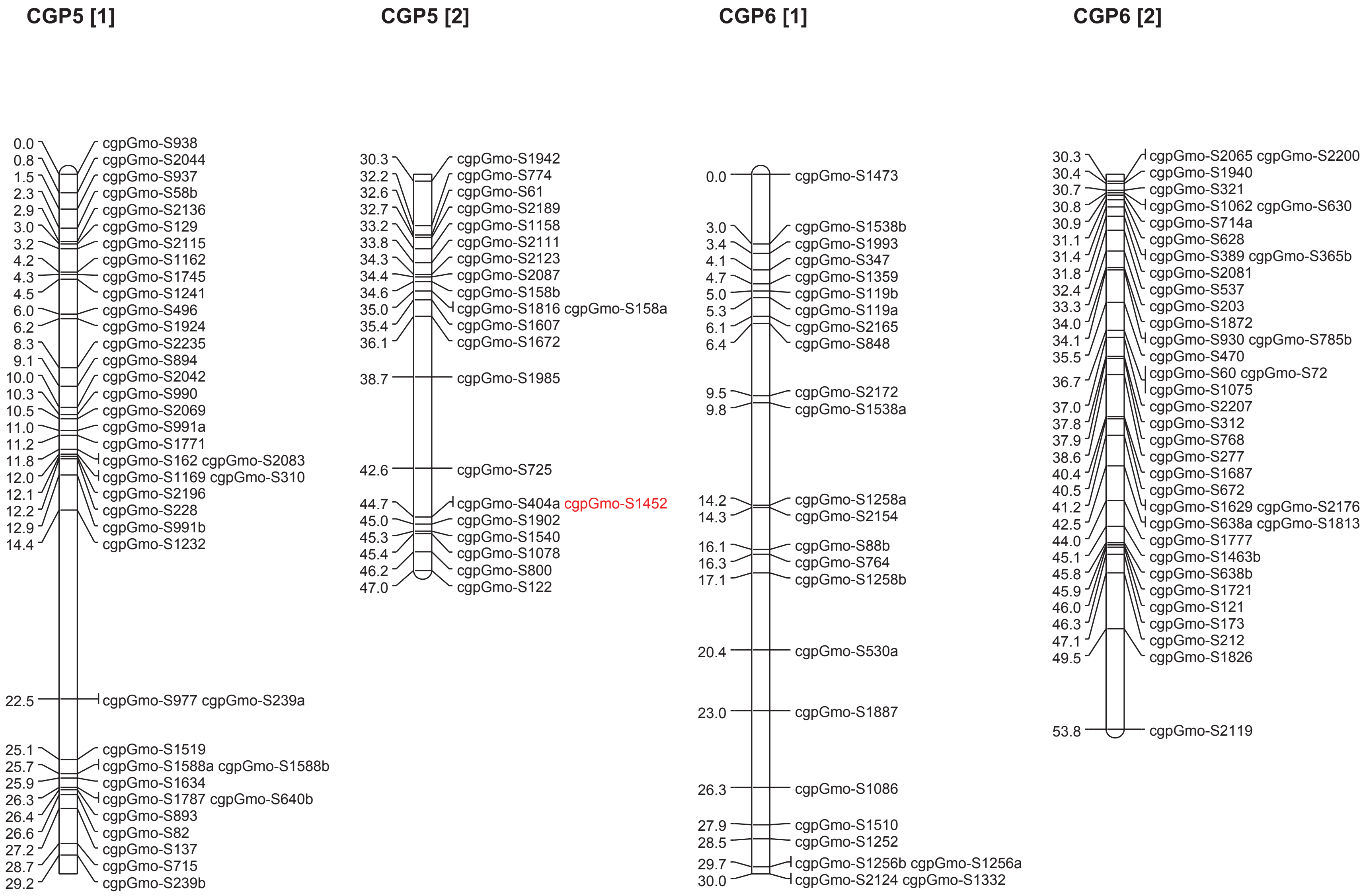
CGP5 [1]
0.0 cgpGmo-S938
0.8 cgpGmo-S2044
1.5 cgpGmo-S937
2.3 cgpGmo-S58b
2.9 cgpGmo-S2136
3.0 cgpGmo-S129
3.2 cgpGmo-S2115
4.2 cgpGmo-S1162
4.3 cgpGmo-S1745
4.5 cgpGmo-S1241
6.0 cgpGmo-S496
6.2 cgpGmo-S1924
8.3 cgpGmo-S2235
9.1 cgpGmo-S894
10.0 cgpGmo-S2042
10.3 cgpGmo-S990
10.5 cgpGmo-S2069
11.0 cgpGmo-S991a
11.2 cgpGmo-S1771
11.8 cgpGmo-S162 cgpGmo-S2083
12.0 cgpGmo-S1169 cgpGmo-S310
12.1 cgpGmo-S2196
12.2 cgpGmo-S228
12.9 cgpGmo-S991b
14.4 cgpGmo-S1232
22.5 cgpGmo-S977 cgpGmo-S239a
25.1 cgpGmo-S1519
25.7 cgpGmo-S1588a cgpGmo-S1588b
25.9 cgpGmo-S1634
26.3 cgpGmo-S1787 cgpGmo-S640b
26.4 cgpGmo-S893
26.6 cgpGmo-S82
27.2 cgpGmo-S137
28.7 cgpGmo-S715
29.2 cgpGmo-S239b
CGP5 [2]
30.3 cgpGmo-S1942
32.2 cgpGmo-S774
32.6 cgpGmo-S61
32.7 cgpGmo-S2189
33.2 cgpGmo-S1158
33.8 cgpGmo-S2111
34.3 cgpGmo-S2123
34.4 cgpGmo-S2087
34.6 cgpGmo-S158b
35.0 cgpGmo-S1816 cgpGmo-S158a
35.4 cgpGmo-S1607
36.1 cgpGmo-S1672
38.7 cgpGmo-S1985
42.6 cgpGmo-S725
44.7 cgpGmo-S404a cgpGmo-S1452
45.0 cgpGmo-S1902
45.3 cgpGmo-S1540
45.4 cgpGmo-S1078
46.2 cgpGmo-S800
47.0 cgpGmo-S122
CGP6 [1]
0.0 cgpGmo-S1473
3.0 cgpGmo-S1538b
3.4 cgpGmo-S1993
4.1 cgpGmo-S347
4.7 cgpGmo-S1359
5.0 cgpGmo-S119b
5.3 cgpGmo-S119a
6.1 cgpGmo-S2165
6.4 cgpGmo-S848
9.5 cgpGmo-S2172
9.8 cgpGmo-S1538a
14.2 cgpGmo-S1258a
14.3 cgpGmo-S2154
16.1 cgpGmo-S88b
16.3 cgpGmo-S764
17.1 cgpGmo-S1258b
20.4 cgpGmo-S530a
23.0 cgpGmo-S1887
26.3 cgpGmo-S1086
27.9 cgpGmo-S1510
28.5 cgpGmo-S1252
29.7 cgpGmo-S1256b cgpGmo-S1256a
30.0 cgpGmo-S2124 cgpGmo-S1332
CGP6 [2]
30.3 cgpGmo-S2065 cgpGmo-S2200
30.4 cgpGmo-S1940
30.7 cgpGmo-S321
30.8 cgpGmo-S1062 cgpGmo-S630
30.9 cgpGmo-S714a
31.1 cgpGmo-S628
31.4 cgpGmo-S389 cgpGmo-S365b
31.8 cgpGmo-S2081
32.4 cgpGmo-S537
33.3 cgpGmo-S203
34.0 cgpGmo-S1872
34.1 cgpGmo-S930 cgpGmo-S785b
35.5 cgpGmo-S470
36.7 cgpGmo-S60 cgpGmo-S72
cgpGmo-S1075
37.0 cgpGmo-S2207
37.8 cgpGmo-S312
37.9 cgpGmo-S768
38.6 cgpGmo-S277
40.4 cgpGmo-S1687
40.5 cgpGmo-S672
41.2 cgpGmo-S1629 cgpGmo-S2176
42.5 cgpGmo-S638a cgpGmo-S1813
44.0 cgpGmo-S1777
45.1 cgpGmo-S1463b
45.8 cgpGmo-S638b
45.9 cgpGmo-S1721
46.0 cgpGmo-S121
46.3 cgpGmo-S173
47.1 cgpGmo-S212
49.5 cgpGmo-S1826
53.8 cgpGmo-S2119

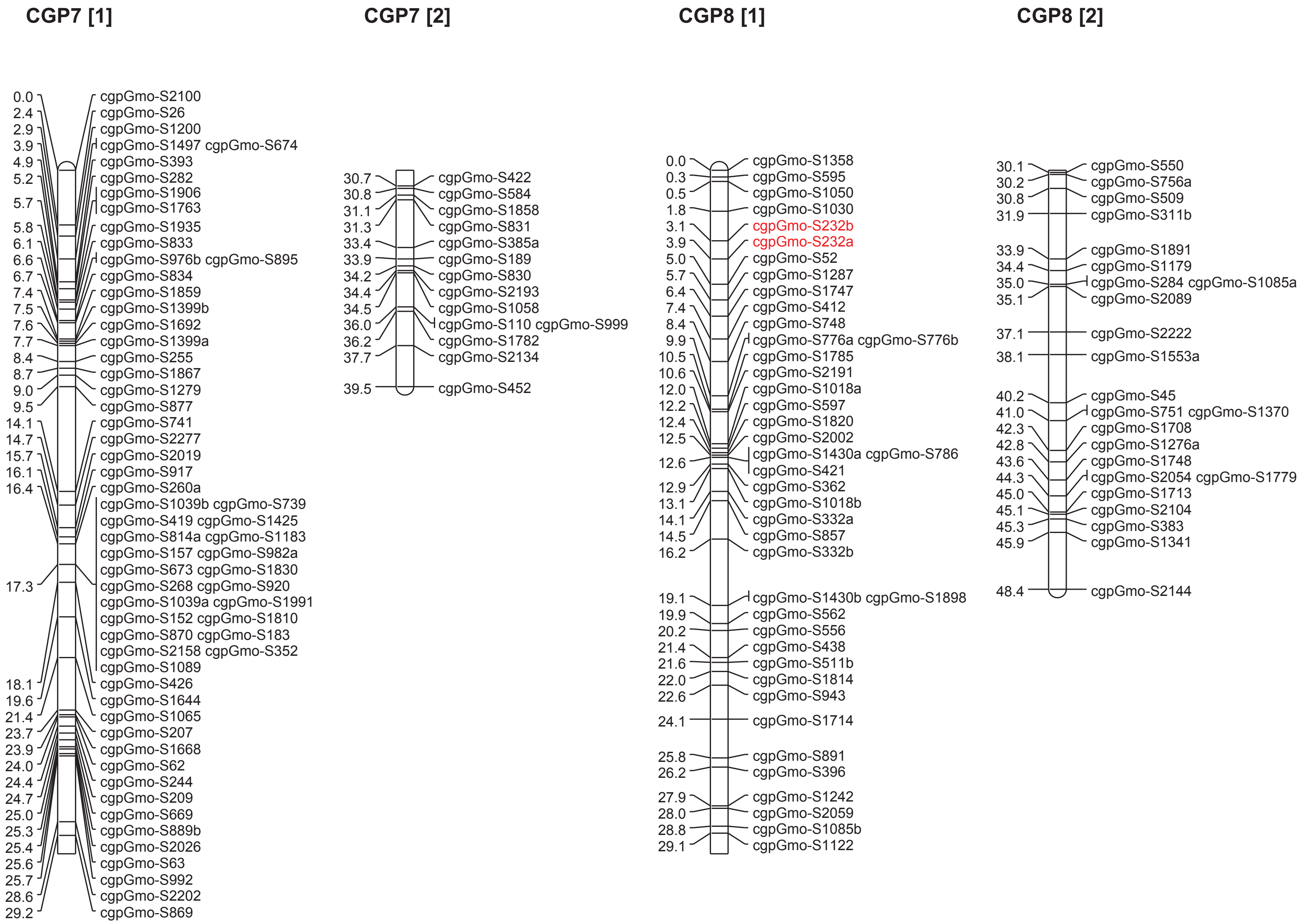
CGP7 [1]
0.0 cgpGmo-S2100
2.4 cgpGmo-S26
2.9 cgpGmo-S1200
3.9 cgpGmo-S1497 cgpGmo-S674
4.9 cgpGmo-S393
5.2 cgpGmo-S282
5.7 cgpGmo-S1906
cgpGmo-S1763
5.8 cgpGmo-S1935
6.1 cgpGmo-S833
6.6 cgpGmo-S976b cgpGmo-S895
6.7 cgpGmo-S834
7.4 cgpGmo-S1859
7.5 cgpGmo-S1399b
7.6 cgpGmo-S1692
7.7 cgpGmo-S1399a
8.4 cgpGmo-S255
8.7 cgpGmo-S1867
9.0 cgpGmo-S1279
9.5 cgpGmo-S877
14.1 cgpGmo-S741
14.7 cgpGmo-S2277
15.7 cgpGmo-S2019
16.1 cgpGmo-S917
16.4 cgpGmo-S260a
cgpGmo-S1039b cgpGmo-S739
cgpGmo-S419 cgpGmo-S1425
cgpGmo-S814a cgpGmo-S1183
cgpGmo-S157 cgpGmo-S982a
cgpGmo-S673 cgpGmo-S1830
17.3 cgpGmo-S268 cgpGmo-S920
cgpGmo-S1039a cgpGmo-S1991
cgpGmo-S152 cgpGmo-S1810
cgpGmo-S870 cgpGmo-S183
cgpGmo-S2158 cgpGmo-S352
cgpGmo-S1089
18.1 cgpGmo-S426
19.6 cgpGmo-S1644
21.4 cgpGmo-S1065
23.7 cgpGmo-S207
23.9 cgpGmo-S1668
24.0 cgpGmo-S62
24.4 cgpGmo-S244
24.7 cgpGmo-S209
25.0 cgpGmo-S669
25.3 cgpGmo-S889b
25.4 cgpGmo-S2026
25.6 cgpGmo-S63
25.7 cgpGmo-S992
28.6 cgpGmo-S2202
29.2 cgpGmo-S869
CGP7 [2]
30.7 cgpGmo-S422
30.8 cgpGmo-S584
31.1 cgpGmo-S1858
31.3 cgpGmo-S831
33.4 cgpGmo-S385a
33.9 cgpGmo-S189
34.2 cgpGmo-S830
34.4 cgpGmo-S2193
34.5 cgpGmo-S1058
36.0 cgpGmo-S110 cgpGmo-S999
36.2 cgpGmo-S1782
37.7 cgpGmo-S2134
39.5 cgpGmo-S452
CGP8 [1]
0.0 cgpGmo-S1358
0.3 cgpGmo-S595
0.5 cgpGmo-S1050
1.8 cgpGmo-S1030
3.1 cgpGmo-S232b
3.9 cgpGmo-S232a
5.0 cgpGmo-S52
5.7 cgpGmo-S1287
6.4 cgpGmo-S1747
7.4 cgpGmo-S412
8.4 cgpGmo-S748
9.9 cgpGmo-S776a cgpGmo-S776b
10.5 cgpGmo-S1785
10.6 cgpGmo-S2191
12.0 cgpGmo-S1018a
12.2 cgpGmo-S597
12.4 cgpGmo-S1820
12.5 cgpGmo-S2002
12.6 cgpGmo-S1430a cgpGmo-S786
cgpGmo-S421
12.9 cgpGmo-S362
13.1 cgpGmo-S1018b
14.1 cgpGmo-S332a
14.5 cgpGmo-S857
16.2 cgpGmo-S332b
19.1 cgpGmo-S1430b cgpGmo-S1898
19.9 cgpGmo-S562
20.2 cgpGmo-S556
21.4 cgpGmo-S438
21.6 cgpGmo-S511b
22.0 cgpGmo-S1814
22.6 cgpGmo-S943
24.1 cgpGmo-S1714
25.8 cgpGmo-S891
26.2 cgpGmo-S396
27.9 cgpGmo-S1242
28.0 cgpGmo-S2059
28.8 cgpGmo-S1085b
29.1 cgpGmo-S1122
CGP8 [2]
30.1 cgpGmo-S550
30.2 cgpGmo-S756a
30.8 cgpGmo-S509
31.9 cgpGmo-S311b
33.9 cgpGmo-S1891
34.4 cgpGmo-S1179
35.0 cgpGmo-S284 cgpGmo-S1085a
35.1 cgpGmo-S2089
37.1 cgpGmo-S2222
38.1 cgpGmo-S1553a
40.2 cgpGmo-S45
41.0 cgpGmo-S751 cgpGmo-S1370
42.3 cgpGmo-S1708
42.8 cgpGmo-S1276a
43.6 cgpGmo-S1748
44.3 cgpGmo-S2054 cgpGmo-S1779
45.0 cgpGmo-S1713
45.1 cgpGmo-S2104
45.3 cgpGmo-S383
45.9 cgpGmo-S1341
48.4 cgpGmo-S2144

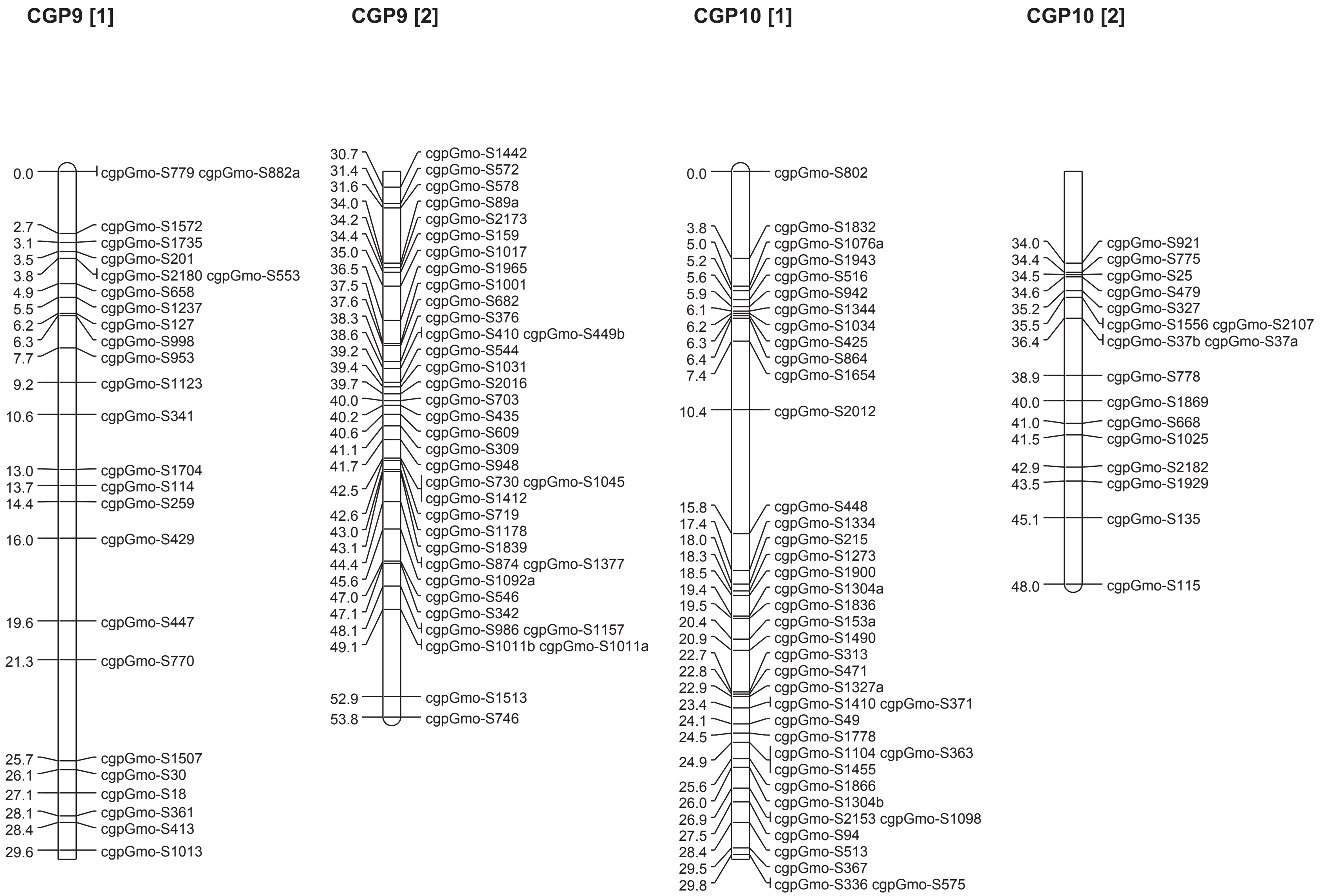
CGP9 [1]
0.0 cgpGmo-S779 cgpGmo-S882a
2.7 cgpGmo-S1572
3.1 cgpGmo-S1735
3.5 cgpGmo-S201
3.8 cgpGmo-S2180 cgpGmo-S553
4.9 cgpGmo-S658
5.5 cgpGmo-S1237
6.2 cgpGmo-S127
6.3 cgpGmo-S998
7.7 cgpGmo-S953
9.2 cgpGmo-S1123
10.6 cgpGmo-S341
13.0 cgpGmo-S1704
13.7 cgpGmo-S114
14.4 cgpGmo-S259
16.0 cgpGmo-S429
19.6 cgpGmo-S447
21.3 cgpGmo-S770
25.7 cgpGmo-S1507
26.1 cgpGmo-S30
27.1 cgpGmo-S18
28.1 cgpGmo-S361
28.4 cgpGmo-S413
29.6 cgpGmo-S1013
CGP9 [2]
30.7 cgpGmo-S1442
31.4 cgpGmo-S572
31.6 cgpGmo-S578
34.0 cgpGmo-S89a
34.2 cgpGmo-S2173
34.4 cgpGmo-S159
35.0 cgpGmo-S1017
36.5 cgpGmo-S1965
37.5 cgpGmo-S1001
37.6 cgpGmo-S682
38.3 cgpGmo-S376
38.6 cgpGmo-S410 cgpGmo-S449b
39.2 cgpGmo-S544
39.4 cgpGmo-S1031
39.7 cgpGmo-S2016
40.0 cgpGmo-S703
40.2 cgpGmo-S435
40.6 cgpGmo-S609
41.1 cgpGmo-S309
41.7 cgpGmo-S948
42.5 cgpGmo-S730 cgpGmo-S1045
cgpGmo-S1412
42.6 cgpGmo-S719
43.0 cgpGmo-S1178
43.1 cgpGmo-S1839
44.4 cgpGmo-S874 cgpGmo-S1377
45.6 cgpGmo-S1092a
47.0 cgpGmo-S546
47.1 cgpGmo-S342
48.1 cgpGmo-S986 cgpGmo-S1157
49.1 cgpGmo-S1011b cgpGmo-S1011a
52.9 cgpGmo-S1513
53.8 cgpGmo-S746
CGP10 [1]
0.0 cgpGmo-S802
3.8 cgpGmo-S1832
5.0 cgpGmo-S1076a
5.2 cgpGmo-S1943
5.6 cgpGmo-S516
5.9 cgpGmo-S942
6.1 cgpGmo-S1344
6.2 cgpGmo-S1034
6.3 cgpGmo-S425
6.4 cgpGmo-S864
7.4 cgpGmo-S1654
10.4 cgpGmo-S2012
15.8 cgpGmo-S448
17.4 cgpGmo-S1334
18.0 cgpGmo-S215
18.3 cgpGmo-S1273
18.5 cgpGmo-S1900
19.4 cgpGmo-S1304a
19.5 cgpGmo-S1836
20.4 cgpGmo-S153a
20.9 cgpGmo-S1490
22.7 cgpGmo-S313
22.8 cgpGmo-S471
22.9 cgpGmo-S1327a
23.4 cgpGmo-S1410 cgpGmo-S371
24.1 cgpGmo-S49
24.5 cgpGmo-S1778
24.9 cgpGmo-S1104 cgpGmo-S363
cgpGmo-S1455
25.6 cgpGmo-S1866
26.0 cgpGmo-S1304b
26.9 cgpGmo-S2153 cgpGmo-S1098
27.5 cgpGmo-S94
28.4 cgpGmo-S513
29.5 cgpGmo-S367
29.8 cgpGmo-S336 cgpGmo-S575
CGP10 [2]
34.0 cgpGmo-S921
34.4 cgpGmo-S775
34.5 cgpGmo-S25
34.6 cgpGmo-S479
35.2 cgpGmo-S327
35.5 cgpGmo-S1556 cgpGmo-S2107
36.4 cgpGmo-S37b cgpGmo-S37a
38.9 cgpGmo-S778
40.0 cgpGmo-S1869
41.0 cgpGmo-S668
41.5 cgpGmo-S1025
42.9 cgpGmo-S2182
43.5 cgpGmo-S1929
45.1 cgpGmo-S135
48.0 cgpGmo-S115

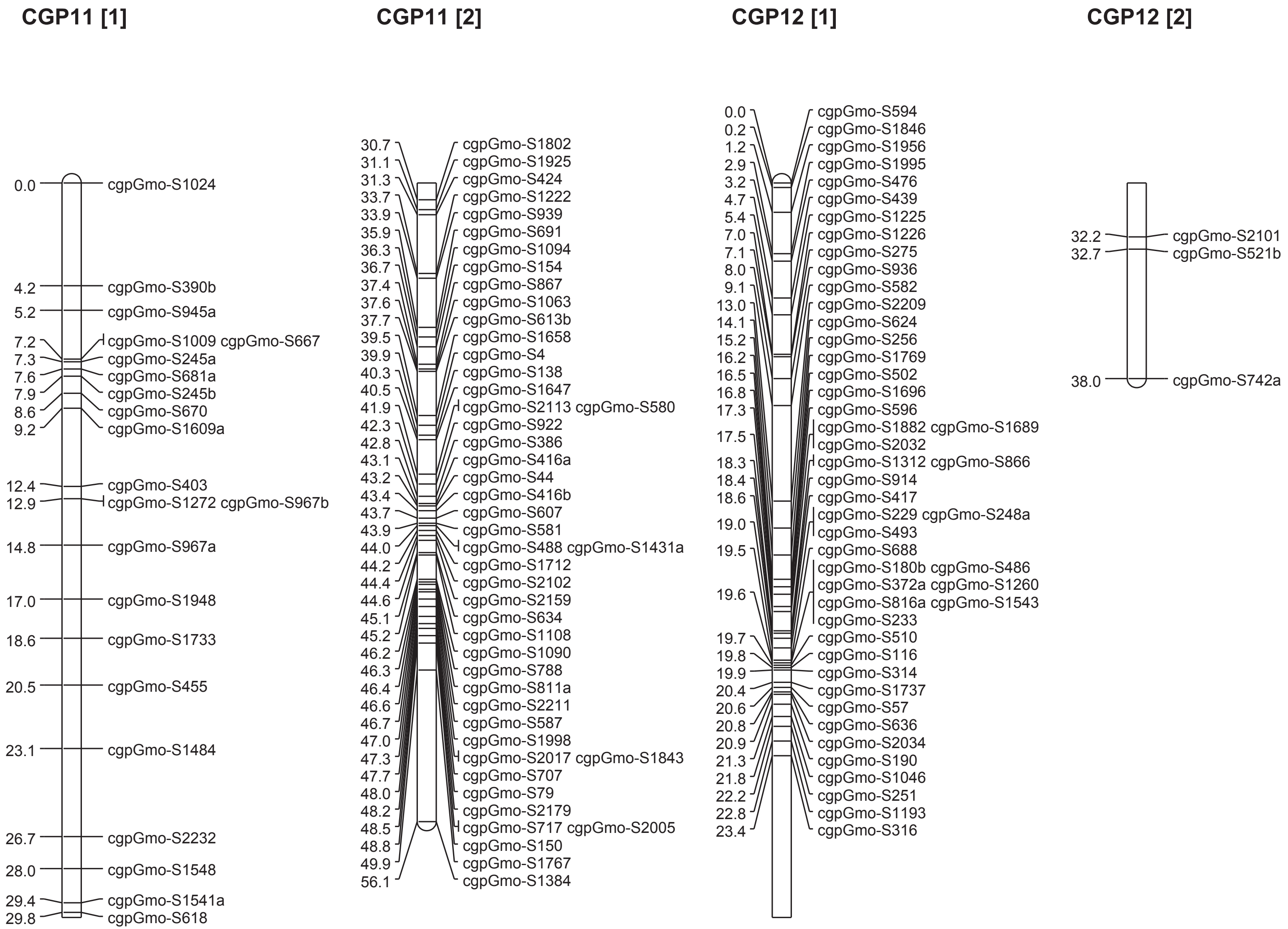
CGP11 [1]
0.0 cgpGmo-S1024
4.2 cgpGmo-S390b
5.2 cgpGmo-S945a
7.2 cgpGmo-S1009 cgpGmo-S667
7.3 cgpGmo-S245a
7.6 cgpGmo-S681a
7.9 cgpGmo-S245b
8.6 cgpGmo-S670
9.2 cgpGmo-S1609a
12.4 cgpGmo-S403
12.9 cgpGmo-S1272 cgpGmo-S967b
14.8 cgpGmo-S967a
17.0 cgpGmo-S1948
18.6 cgpGmo-S1733
20.5 cgpGmo-S455
23.1 cgpGmo-S1484
26.7 cgpGmo-S2232
28.0 cgpGmo-S1548
29.4 cgpGmo-S1541a
29.8 cgpGmo-S618
CGP11 [2]
30.7 cgpGmo-S1802
31.1 cgpGmo-S1925
31.3 cgpGmo-S424
33.7 cgpGmo-S1222
33.9 cgpGmo-S939
35.9 cgpGmo-S691
36.3 cgpGmo-S1094
36.7 cgpGmo-S154
37.4 cgpGmo-S867
37.6 cgpGmo-S1063
37.7 cgpGmo-S613b
39.5 cgpGmo-S1658
39.9 cgpGmo-S4
40.3 cgpGmo-S138
40.5 cgpGmo-S1647
41.9 cgpGmo-S2113 cgpGmo-S580
42.3 cgpGmo-S922
42.8 cgpGmo-S386
43.1 cgpGmo-S416a
43.2 cgpGmo-S44
43.4 cgpGmo-S416b
43.7 cgpGmo-S607
43.9 cgpGmo-S581
44.0 cgpGmo-S488 cgpGmo-S1431a
44.2 cgpGmo-S1712
44.4 cgpGmo-S2102
44.6 cgpGmo-S2159
45.1 cgpGmo-S634
45.2 cgpGmo-S1108
46.2 cgpGmo-S1090
46.3 cgpGmo-S788
46.4 cgpGmo-S811a
46.6 cgpGmo-S2211
46.7 cgpGmo-S587
47.0 cgpGmo-S1998
47.3 cgpGmo-S2017 cgpGmo-S1843
47.7 cgpGmo-S707
48.0 cgpGmo-S79
48.2 cgpGmo-S2179
48.5 cgpGmo-S717 cgpGmo-S2005
48.8 cgpGmo-S150
49.9 cgpGmo-S1767
56.1 cgpGmo-S1384
CGP12 [1]
0.0 cgpGmo-S594
0.2 cgpGmo-S1846
1.2 cgpGmo-S1956
2.9 cgpGmo-S1995
3.2 cgpGmo-S476
4.7 cgpGmo-S439
5.4 cgpGmo-S1225
7.0 cgpGmo-S1226
7.1 cgpGmo-S275
8.0 cgpGmo-S936
9.1 cgpGmo-S582
13.0 cgpGmo-S2209
14.1 cgpGmo-S624
15.2 cgpGmo-S256
16.2 cgpGmo-S1769
16.5 cgpGmo-S502
16.8 cgpGmo-S1696
17.3 cgpGmo-S596
17.5 cgpGmo-S1882 cgpGmo-S1689
cgpGmo-S2032
18.3 cgpGmo-S1312 cgpGmo-S866
18.4 cgpGmo-S914
18.6 cgpGmo-S417
19.0 cgpGmo-S229 cgpGmo-S248a
cgpGmo-S493
19.5 cgpGmo-S688
19.6 cgpGmo-S180b cgpGmo-S486
cgpGmo-S372a cgpGmo-S1260
cgpGmo-S816a cgpGmo-S1543
cgpGmo-S233
19.7 cgpGmo-S510
19.8 cgpGmo-S116
19.9 cgpGmo-S314
20.4 cgpGmo-S1737
20.6 cgpGmo-S57
20.8 cgpGmo-S636
20.9 cgpGmo-S2034
21.3 cgpGmo-S190
21.8 cgpGmo-S1046
22.2 cgpGmo-S251
22.8 cgpGmo-S1193
23.4 cgpGmo-S316
CGP12 [2]
32.2 cgpGmo-S2101
32.7 cgpGmo-S521b
38.0 cgpGmo-S742a

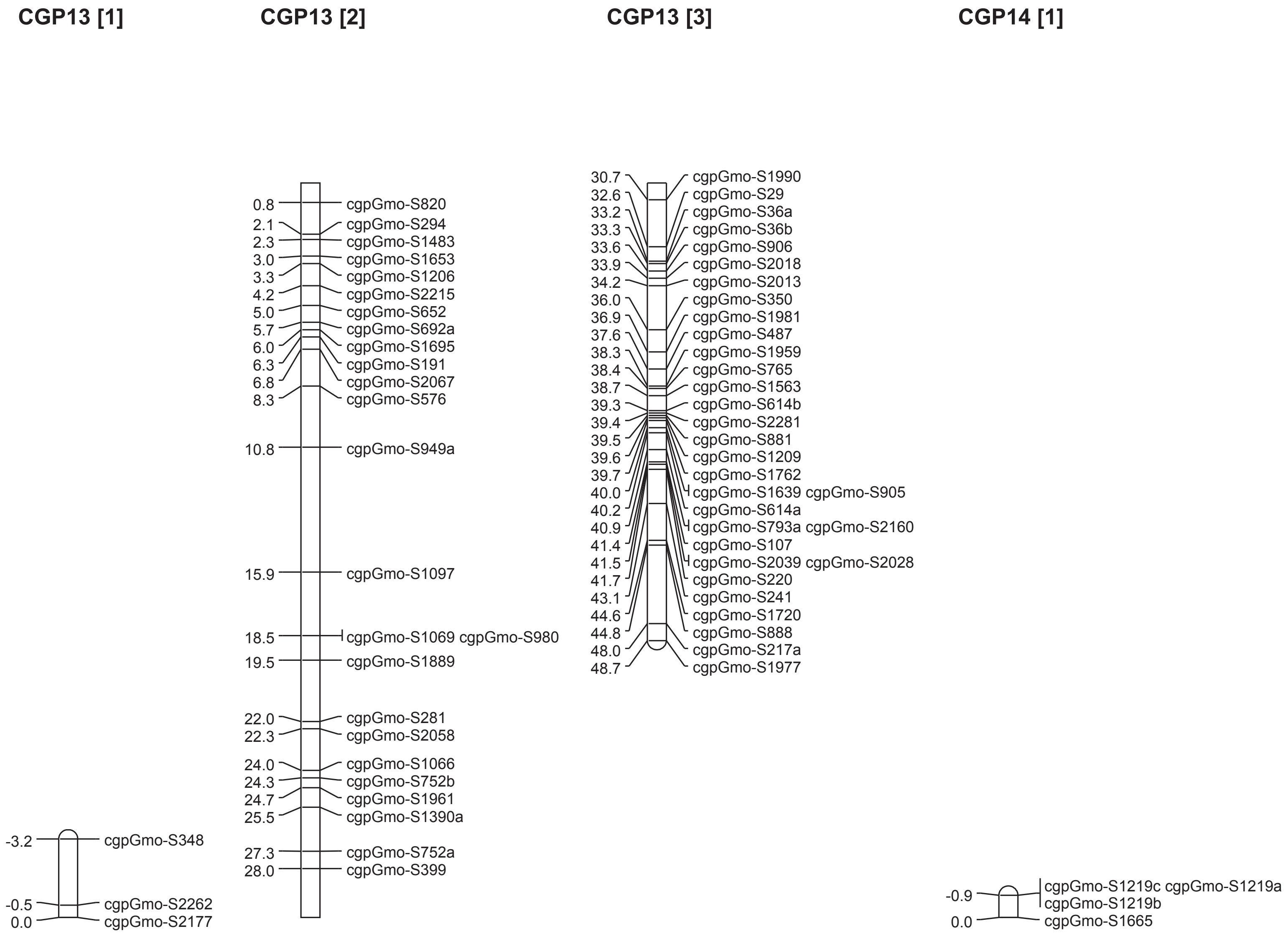
CGP13 [1]
-3.2 cgpGmo-S348
-0.5 cgpGmo-S2262
0.0 cgpGmo-S2177
CGP13 [2]
0.8 cgpGmo-S820
2.1 cgpGmo-S294
2.3 cgpGmo-S1483
3.0 cgpGmo-S1653
3.3 cgpGmo-S1206
4.2 cgpGmo-S2215
5.0 cgpGmo-S652
5.7 cgpGmo-S692a
6.0 cgpGmo-S1695
6.3 cgpGmo-S191
6.8 cgpGmo-S2067
8.3 cgpGmo-S576
10.8 cgpGmo-S949a
15.9 cgpGmo-S1097
18.5 cgpGmo-S1069 cgpGmo-S980
19.5 cgpGmo-S1889
22.0 cgpGmo-S281
22.3 cgpGmo-S2058
24.0 cgpGmo-S1066
24.3 cgpGmo-S752b
24.7 cgpGmo-S1961
25.5 cgpGmo-S1390a
27.3 cgpGmo-S752a
28.0 cgpGmo-S399
CGP13 [3]
30.7 cgpGmo-S1990
32.6 cgpGmo-S29
33.2 cgpGmo-S36a
33.3 cgpGmo-S36b
33.6 cgpGmo-S906
33.9 cgpGmo-S2018
34.2 cgpGmo-S2013
36.0 cgpGmo-S350
36.9 cgpGmo-S1981
37.6 cgpGmo-S487
38.3 cgpGmo-S1959
38.4 cgpGmo-S765
38.7 cgpGmo-S1563
39.3 cgpGmo-S614b
39.4 cgpGmo-S2281
39.5 cgpGmo-S881
39.6 cgpGmo-S1209
39.7 cgpGmo-S1762
40.0 cgpGmo-S1639 cgpGmo-S905
40.2 cgpGmo-S614a
40.9 cgpGmo-S793a cgpGmo-S2160
41.4 cgpGmo-S107
41.5 cgpGmo-S2039 cgpGmo-S2028
41.7 cgpGmo-S220
43.1 cgpGmo-S241
44.6 cgpGmo-S1720
44.8 cgpGmo-S888
48.0 cgpGmo-S217a
48.7 cgpGmo-S1977
CGP14 [1]
-0.9 cgpGmo-S1219c cgpGmo-S1219a
cgpGmo-S1219b
0.0 cgpGmo-S1665

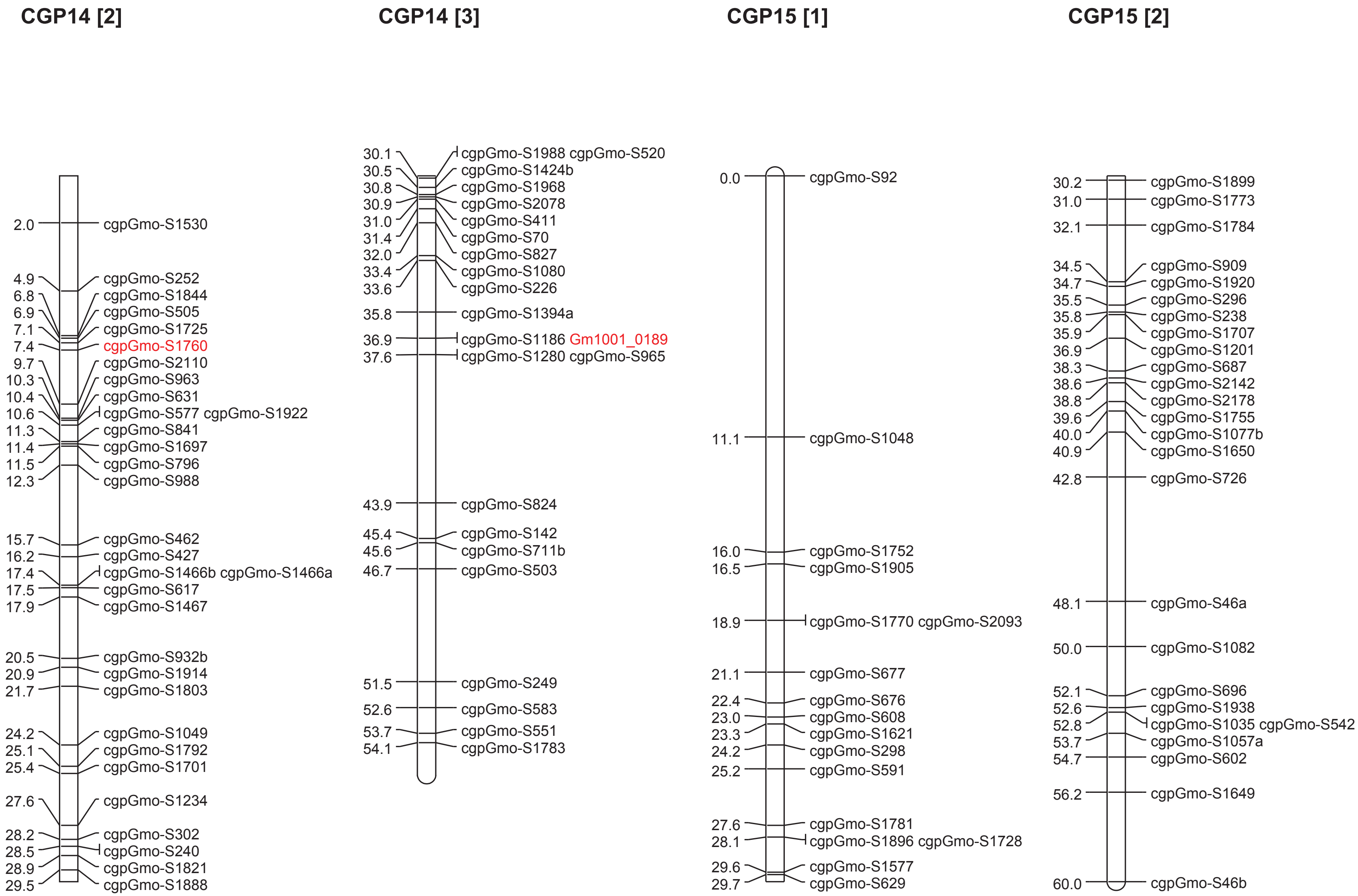
CGP14 [2]
2.0 cgpGmo-S1530
4.9 cgpGmo-S252
6.8 cgpGmo-S1844
6.9 cgpGmo-S505
7.1 cgpGmo-S1725
7.4 cgpGmo-S1760
9.7 cgpGmo-S2110
10.3 cgpGmo-S963
10.4 cgpGmo-S631
10.6 cgpGmo-S577 cgpGmo-S1922
11.3 cgpGmo-S841
11.4 cgpGmo-S1697
11.5 cgpGmo-S796
12.3 cgpGmo-S988
15.7 cgpGmo-S462
16.2 cgpGmo-S427
17.4 cgpGmo-S1466b cgpGmo-S1466a
17.5 cgpGmo-S617
17.9 cgpGmo-S1467
20.5 cgpGmo-S932b
20.9 cgpGmo-S1914
21.7 cgpGmo-S1803
24.2 cgpGmo-S1049
25.1 cgpGmo-S1792
25.4 cgpGmo-S1701
27.6 cgpGmo-S1234
28.2 cgpGmo-S302
28.5 cgpGmo-S240
28.9 cgpGmo-S1821
29.5 cgpGmo-S1888
CGP14 [3]
30.1 cgpGmo-S1988 cgpGmo-S520
30.5 cgpGmo-S1424b
30.8 cgpGmo-S1968
30.9 cgpGmo-S2078
31.0 cgpGmo-S411
31.4 cgpGmo-S70
32.0 cgpGmo-S827
33.4 cgpGmo-S1080
33.6 cgpGmo-S226
35.8 cgpGmo-S1394a
36.9 cgpGmo-S1186 Gm1001_0189
37.6 cgpGmo-S1280 cgpGmo-S965
43.9 cgpGmo-S824
45.4 cgpGmo-S142
45.6 cgpGmo-S711b
46.7 cgpGmo-S503
51.5 cgpGmo-S249
52.6 cgpGmo-S583
53.7 cgpGmo-S551
54.1 cgpGmo-S1783
CGP15 [1]
0.0 cgpGmo-S92
11.1 cgpGmo-S1048
16.0 cgpGmo-S1752
16.5 cgpGmo-S1905
18.9 cgpGmo-S1770 cgpGmo-S2093
21.1 cgpGmo-S677
22.4 cgpGmo-S676
23.0 cgpGmo-S608
23.3 cgpGmo-S1621
24.2 cgpGmo-S298
25.2 cgpGmo-S591
27.6 cgpGmo-S1781
28.1 cgpGmo-S1896 cgpGmo-S1728
29.6 cgpGmo-S1577
29.7 cgpGmo-S629
CGP15 [2]
30.2 cgpGmo-S1899
31.0 cgpGmo-S1773
32.1 cgpGmo-S1784
34.5 cgpGmo-S909
34.7 cgpGmo-S1920
35.5 cgpGmo-S296
35.8 cgpGmo-S238
35.9 cgpGmo-S1707
36.9 cgpGmo-S1201
38.3 cgpGmo-S687
38.6 cgpGmo-S2142
38.8 cgpGmo-S2178
39.6 cgpGmo-S1755
40.0 cgpGmo-S1077b
40.9 cgpGmo-S1650
42.8 cgpGmo-S726
48.1 cgpGmo-S46a
50.0 cgpGmo-S1082
52.1 cgpGmo-S696
52.6 cgpGmo-S1938
52.8 cgpGmo-S1035 cgpGmo-S542
53.7 cgpGmo-S1057a
54.7 cgpGmo-S602
56.2 cgpGmo-S1649
60.0 cgpGmo-S46b

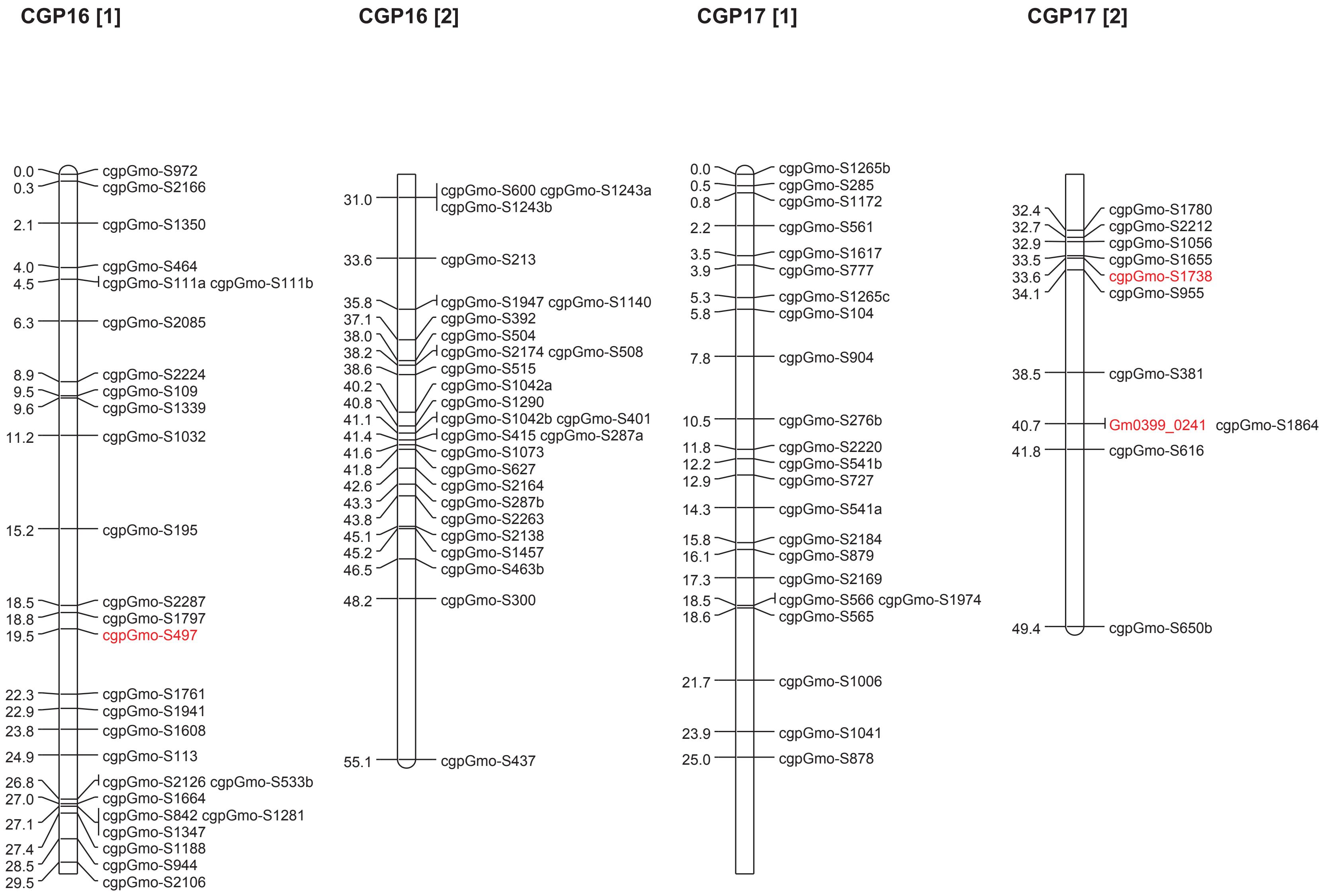
CGP16 [1]
0.0 cgpGmo-S972
0.3 cgpGmo-S2166
2.1 cgpGmo-S1350
4.0 cgpGmo-S464
4.5 cgpGmo-S111a cgpGmo-S111b
6.3 cgpGmo-S2085
8.9 cgpGmo-S2224
9.5 cgpGmo-S109
9.6 cgpGmo-S1339
11.2 cgpGmo-S1032
15.2 cgpGmo-S195
18.5 cgpGmo-S2287
18.8 cgpGmo-S1797
19.5 cgpGmo-S497
22.3 cgpGmo-S1761
22.9 cgpGmo-S1941
23.8 cgpGmo-S1608
24.9 cgpGmo-S113
26.8 cgpGmo-S2126 cgpGmo-S533b
27.0 cgpGmo-S1664
27.1 cgpGmo-S842 cgpGmo-S1281
cgpGmo-S1347
27.4 cgpGmo-S1188
28.5 cgpGmo-S944
29.5 cgpGmo-S2106
CGP16 [2]
31.0 cgpGmo-S600 cgpGmo-S1243a
cgpGmo-S1243b
33.6 cgpGmo-S213
35.8 cgpGmo-S1947 cgpGmo-S1140
37.1 cgpGmo-S392
38.0 cgpGmo-S504
38.2 cgpGmo-S2174 cgpGmo-S508
38.6 cgpGmo-S515
40.2 cgpGmo-S1042a
40.8 cgpGmo-S1290
41.1 cgpGmo-S1042b cgpGmo-S401
41.4 cgpGmo-S415 cgpGmo-S287a
41.6 cgpGmo-S1073
41.8 cgpGmo-S627
42.6 cgpGmo-S2164
43.3 cgpGmo-S287b
43.8 cgpGmo-S2263
45.1 cgpGmo-S2138
45.2 cgpGmo-S1457
46.5 cgpGmo-S463b
48.2 cgpGmo-S300
55.1 cgpGmo-S437
CGP17 [1]
0.0 cgpGmo-S1265b
0.5 cgpGmo-S285
0.8 cgpGmo-S1172
2.2 cgpGmo-S561
3.5 cgpGmo-S1617
3.9 cgpGmo-S777
5.3 cgpGmo-S1265c
5.8 cgpGmo-S104
7.8 cgpGmo-S904
10.5 cgpGmo-S276b
11.8 cgpGmo-S2220
12.2 cgpGmo-S541b
12.9 cgpGmo-S727
14.3 cgpGmo-S541a
15.8 cgpGmo-S2184
16.1 cgpGmo-S879
17.3 cgpGmo-S2169
18.5 cgpGmo-S566 cgpGmo-S1974
18.6 cgpGmo-S565
21.7 cgpGmo-S1006
23.9 cgpGmo-S1041
25.0 cgpGmo-S878
CGP17 [2]
32.4 cgpGmo-S1780
32.7 cgpGmo-S2212
32.9 cgpGmo-S1056
33.5 cgpGmo-S1655
33.6 cgpGmo-S1738
34.1 cgpGmo-S955
38.5 cgpGmo-S381
40.7 Gm0399_0241 cgpGmo-S1864
41.8 cgpGmo-S616
49.4 cgpGmo-S650b

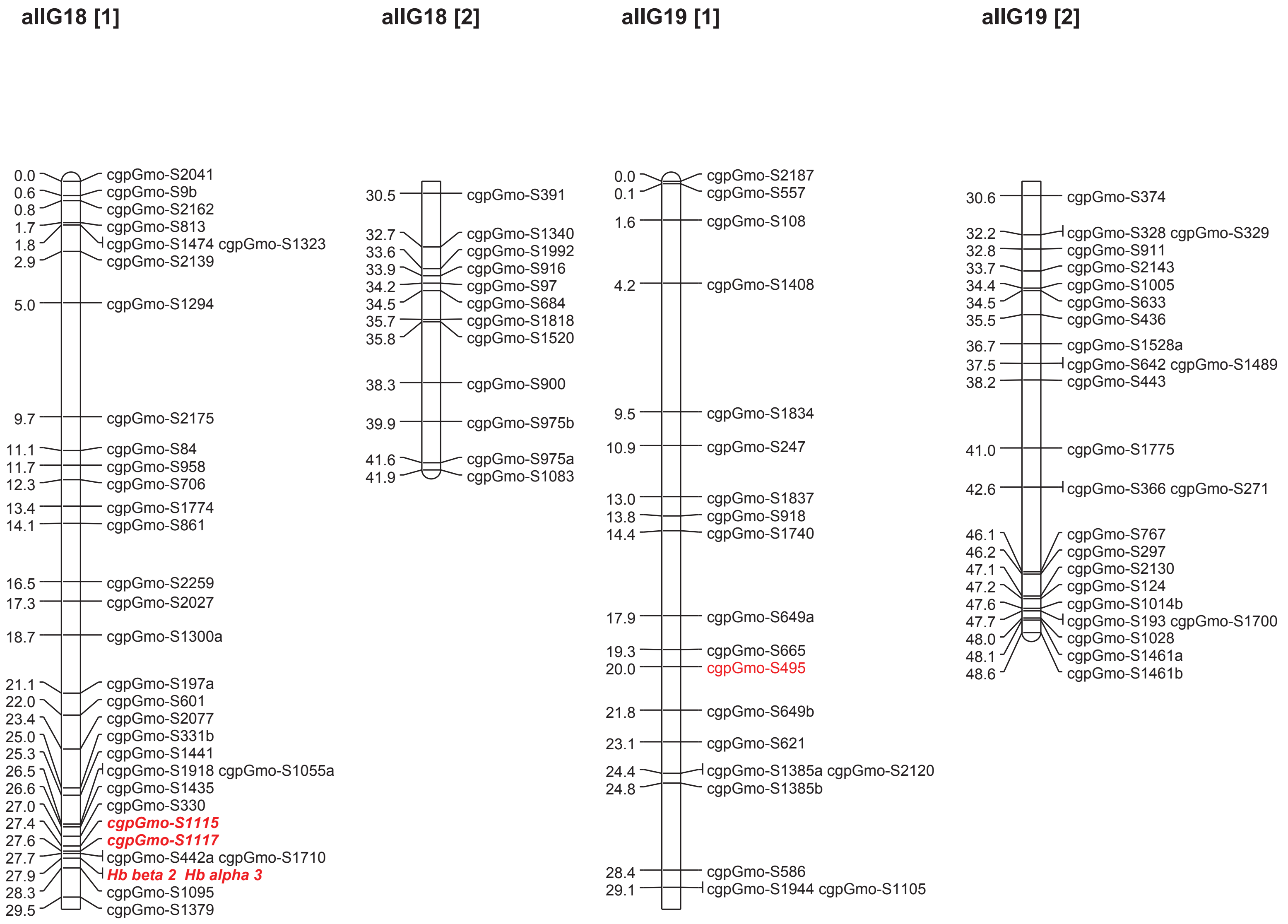
alIG18 [1]
0.0 cgpGmo-S2041
0.6 cgpGmo-S9b
0.8 cgpGmo-S2162
1.7 cgpGmo-S813
1.8 cgpGmo-S1474 cgpGmo-S1323
2.9 cgpGmo-S2139
5.0 cgpGmo-S1294
9.7 cgpGmo-S2175
11.1 cgpGmo-S84
11.7 cgpGmo-S958
12.3 cgpGmo-S706
13.4 cgpGmo-S1774
14.1 cgpGmo-S861
16.5 cgpGmo-S2259
17.3 cgpGmo-S2027
18.7 cgpGmo-S1300a
21.1 cgpGmo-S197a
22.0 cgpGmo-S601
23.4 cgpGmo-S2077
25.0 cgpGmo-S331b
25.3 cgpGmo-S1441
26.5 cgpGmo-S1918 cgpGmo-S1055a
26.6 cgpGmo-S1435
27.0 cgpGmo-S330
27.4 cgpGmo-S1115
27.6 cgpGmo-S1117
27.7 cgpGmo-S442a cgpGmo-S1710
27.9 Hb beta 2 Hb alpha 3
28.3 cgpGmo-S1095
29.5 cgpGmo-S1379
alIG18 [2]
30.5 cgpGmo-S391
32.7 cgpGmo-S1340
33.6 cgpGmo-S1992
33.9 cgpGmo-S916
34.2 cgpGmo-S97
34.5 cgpGmo-S684
35.7 cgpGmo-S1818
35.8 cgpGmo-S1520
38.3 cgpGmo-S900
39.9 cgpGmo-S975b
41.6 cgpGmo-S975a
41.9 cgpGmo-S1083
alIG19 [1]
0.0 cgpGmo-S2187
0.1 cgpGmo-S557
1.6 cgpGmo-S108
4.2 cgpGmo-S1408
9.5 cgpGmo-S1834
10.9 cgpGmo-S247
13.0 cgpGmo-S1837
13.8 cgpGmo-S918
14.4 cgpGmo-S1740
17.9 cgpGmo-S649a
19.3 cgpGmo-S665
20.0 cgpGmo-S495
21.8 cgpGmo-S649b
23.1 cgpGmo-S621
24.4 cgpGmo-S1385a cgpGmo-S2120
24.8 cgpGmo-S1385b
28.4 cgpGmo-S586
29.1 cgpGmo-S1944 cgpGmo-S1105
alIG19 [2]
30.6 cgpGmo-S374
32.2 cgpGmo-S328 cgpGmo-S329
32.8 cgpGmo-S911
33.7 cgpGmo-S2143
34.4 cgpGmo-S1005
34.5 cgpGmo-S633
35.5 cgpGmo-S436
36.7 cgpGmo-S1528a
37.5 cgpGmo-S642 cgpGmo-S1489
38.2 cgpGmo-S443
41.0 cgpGmo-S1775
42.6 cgpGmo-S366 cgpGmo-S271
46.1 cgpGmo-S767
46.2 cgpGmo-S297
47.1 cgpGmo-S2130
47.2 cgpGmo-S124
47.6 cgpGmo-S1014b
47.7 cgpGmo-S193 cgpGmo-S1700
48.0 cgpGmo-S1028
48.1 cgpGmo-S1461a
48.6 cgpGmo-S1461b

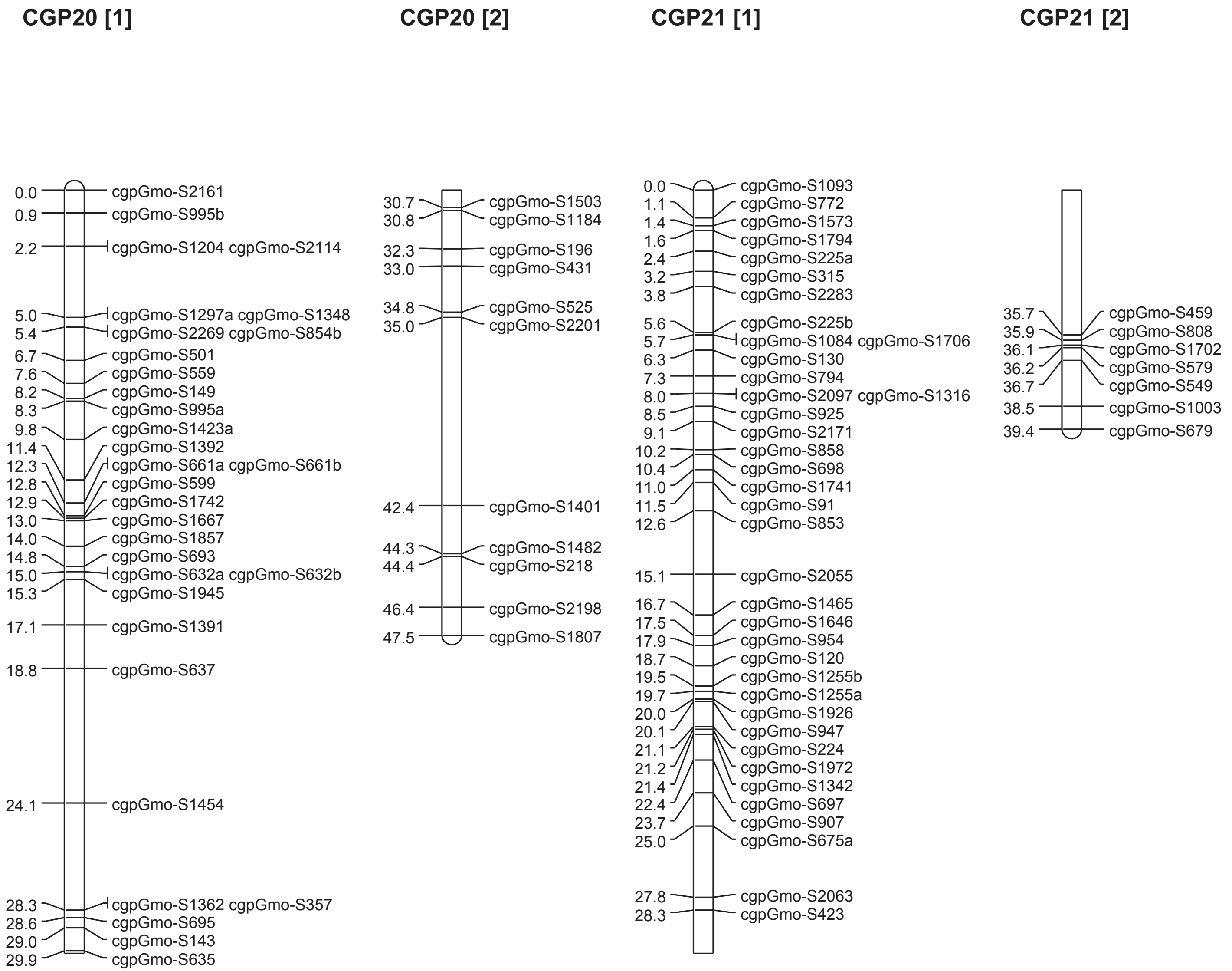
CGP20 [1]
0.0 cgpGmo-S2161
0.9 cgpGmo-S995b
2.2 cgpGmo-S1204 cgpGmo-S2114
5.0 cgpGmo-S1297a cgpGmo-S1348
5.4 cgpGmo-S2269 cgpGmo-S854b
6.7 cgpGmo-S501
7.6 cgpGmo-S559
8.2 cgpGmo-S149
8.3 cgpGmo-S995a
9.8 cgpGmo-S1423a
11.4 cgpGmo-S1392
12.3 cgpGmo-S661a cgpGmo-S661b
12.8 cgpGmo-S599
12.9 cgpGmo-S1742
13.0 cgpGmo-S1667
14.0 cgpGmo-S1857
14.8 cgpGmo-S693
15.0 cgpGmo-S632a cgpGmo-S632b
15.3 cgpGmo-S1945
17.1 cgpGmo-S1391
18.8 cgpGmo-S637
24.1 cgpGmo-S1454
28.3 cgpGmo-S1362 cgpGmo-S357
28.6 cgpGmo-S695
29.0 cgpGmo-S143
29.9 cgpGmo-S635
CGP20 [2]
30.7 cgpGmo-S1503
30.8 cgpGmo-S1184
32.3 cgpGmo-S196
33.0 cgpGmo-S431
34.8 cgpGmo-S525
35.0 cgpGmo-S2201
42.4 cgpGmo-S1401
44.3 cgpGmo-S1482
44.4 cgpGmo-S218
46.4 cgpGmo-S2198
47.5 cgpGmo-S1807
CGP21 [1]
0.0 cgpGmo-S1093
1.1 cgpGmo-S772
1.4 cgpGmo-S1573
1.6 cgpGmo-S1794
2.4 cgpGmo-S225a
3.2 cgpGmo-S315
3.8 cgpGmo-S2283
5.6 cgpGmo-S225b
5.7 cgpGmo-S1084 cgpGmo-S1706
6.3 cgpGmo-S130
7.3 cgpGmo-S794
8.0 cgpGmo-S2097 cgpGmo-S1316
8.5 cgpGmo-S925
9.1 cgpGmo-S2171
10.2 cgpGmo-S858
10.4 cgpGmo-S698
11.0 cgpGmo-S1741
11.5 cgpGmo-S91
12.6 cgpGmo-S853
15.1 cgpGmo-S2055
16.7 cgpGmo-S1465
17.5 cgpGmo-S1646
17.9 cgpGmo-S954
18.7 cgpGmo-S120
19.5 cgpGmo-S1255b
19.7 cgpGmo-S1255a
20.0 cgpGmo-S1926
20.1 cgpGmo-S947
21.1 cgpGmo-S224
21.2 cgpGmo-S1972
21.4 cgpGmo-S1342
22.4 cgpGmo-S697
23.7 cgpGmo-S907
25.0 cgpGmo-S675a
27.8 cgpGmo-S2063
28.3 cgpGmo-S423
CGP21 [2]
35.7 cgpGmo-S459
35.9 cgpGmo-S808
36.1 cgpGmo-S1702
36.2 cgpGmo-S579
36.7 cgpGmo-S549
38.5 cgpGmo-S1003
39.4 cgpGmo-S679

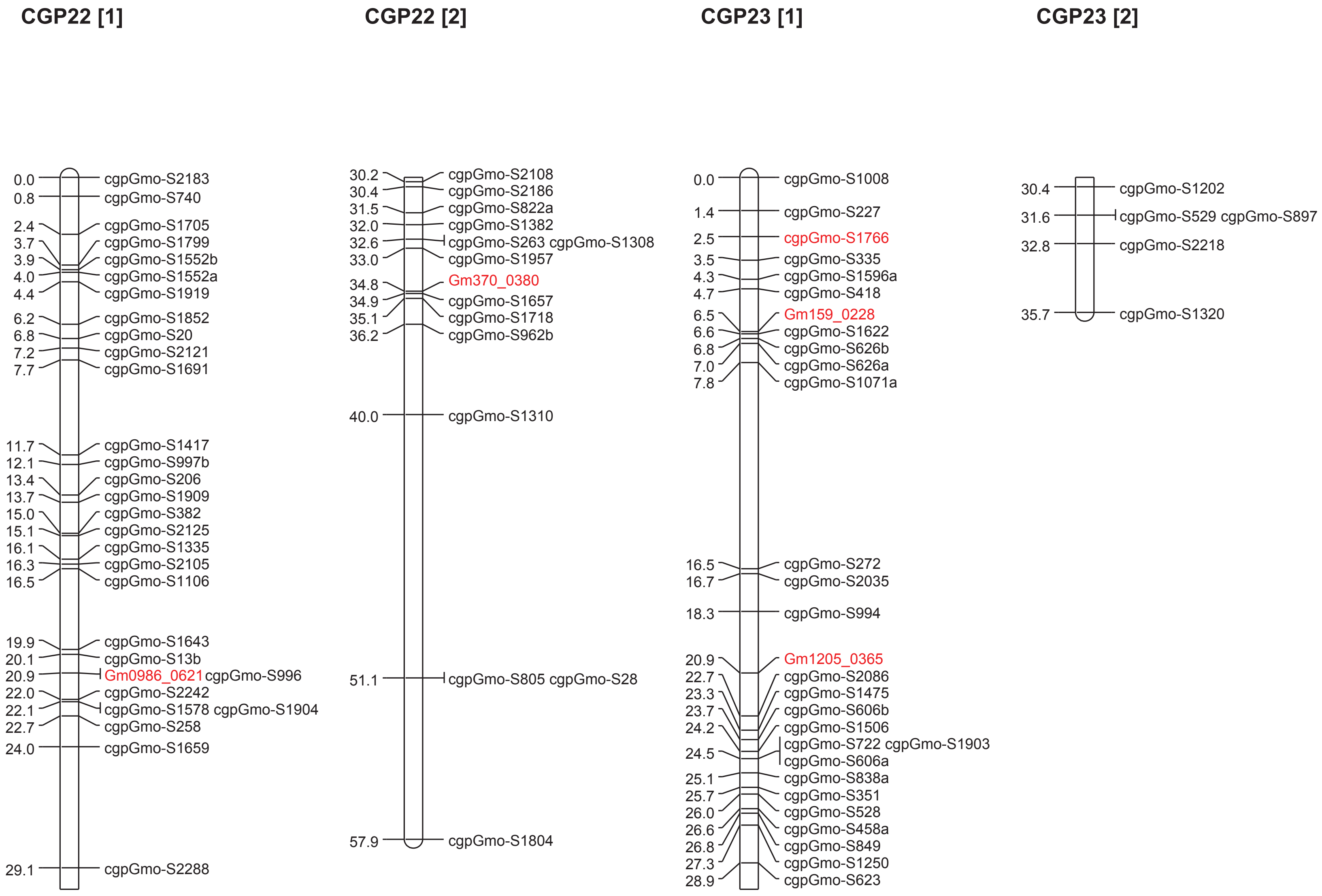
CGP22 [1]
0.0 cgpGmo-S2183
0.8 cgpGmo-S740
2.4 cgpGmo-S1705
3.7 cgpGmo-S1799
3.9 cgpGmo-S1552b
4.0 cgpGmo-S1552a
4.4 cgpGmo-S1919
6.2 cgpGmo-S1852
6.8 cgpGmo-S20
7.2 cgpGmo-S2121
7.7 cgpGmo-S1691
11.7 cgpGmo-S1417
12.1 cgpGmo-S997b
13.4 cgpGmo-S206
13.7 cgpGmo-S1909
15.0 cgpGmo-S382
15.1 cgpGmo-S2125
16.1 cgpGmo-S1335
16.3 cgpGmo-S2105
16.5 cgpGmo-S1106
19.9 cgpGmo-S1643
20.1 cgpGmo-S13b
20.9 Gm0986_0621 cgpGmo-S996
22.0 cgpGmo-S2242
22.1 cgpGmo-S1578 cgpGmo-S1904
22.7 cgpGmo-S258
24.0 cgpGmo-S1659
29.1 cgpGmo-S2288
CGP22 [2]
30.2 cgpGmo-S2108
30.4 cgpGmo-S2186
31.5 cgpGmo-S822a
32.0 cgpGmo-S1382
32.6 cgpGmo-S263 cgpGmo-S1308
33.0 cgpGmo-S1957
34.8 Gm370_0380
34.9 cgpGmo-S1657
35.1 cgpGmo-S1718
36.2 cgpGmo-S962b
40.0 cgpGmo-S1310
51.1 cgpGmo-S805 cgpGmo-S28
57.9 cgpGmo-S1804
CGP23 [1]
0.0 cgpGmo-S1008
1.4 cgpGmo-S227
2.5 cgpGmo-S1766
3.5 cgpGmo-S335
4.3 cgpGmo-S1596a
4.7 cgpGmo-S418
6.5 Gm159_0228
6.6 cgpGmo-S1622
6.8 cgpGmo-S626b
7.0 cgpGmo-S626a
7.8 cgpGmo-S1071a
16.5 cgpGmo-S272
16.7 cgpGmo-S2035
18.3 cgpGmo-S994
20.9 Gm1205_0365
22.7 cgpGmo-S2086
23.3 cgpGmo-S1475
23.7 cgpGmo-S606b
24.2 cgpGmo-S1506
24.5 cgpGmo-S722 cgpGmo-S1903
cgpGmo-S606a
25.1 cgpGmo-S838a
25.7 cgpGmo-S351
26.0 cgpGmo-S528
26.6 cgpGmo-S458a
26.8 cgpGmo-S849
27.3 cgpGmo-S1250
28.9 cgpGmo-S623
CGP23 [2]
30.4 cgpGmo-S1202
31.6 cgpGmo-S529 cgpGmo-S897
32.8 cgpGmo-S2218
35.7 cgpGmo-S1320
